# Innate and secondary humoral responses are improved by increasing the time between MVA vaccine immunizations

**DOI:** 10.1038/s41541-020-0175-8

**Published:** 2020-03-19

**Authors:** Jean-Louis Palgen, Nicolas Tchitchek, André Rodriguez-Pozo, Quentin Jouhault, Hadjer Abdelhouahab, Nathalie Dereuddre-Bosquet, Vanessa Contreras, Frédéric Martinon, Antonio Cosma, Yves Lévy, Roger Le Grand, Anne-Sophie Beignon

**Affiliations:** 1 CEA—Université Paris Sud 11—INSERM U1184, Immunology of Viral Infections and Autoimmune Diseases, IDMIT Department, IBFJ, 92265 Fontenay-aux-Roses, France; 2 Vaccine Research Institute, Henri Mondor Hospital, 94010 Créteil, France; 3 Institut Mondor de Recherche Biomédicale—INSERM U955, Eq.16, 94010 Créteil, France

**Keywords:** Innate immunity, Vaccines

## Abstract

Comprehending the mechanisms behind the impact of vaccine regimens on immunity is critical for improving vaccines. Indeed, the time-interval between immunizations may influence B and T cells, as well as innate responses. We compared two vaccine schedules using cynomolgus macaques immunized with an attenuated vaccinia virus. Two subcutaneous injections 2 weeks apart led to an impaired secondary antibody response and similar innate myeloid responses to both immunizations. In contrast, a delayed boost (2 months) improved the quality of the antibody response and involved more activated/mature innate cells, induced late after the prime and responding to the recall. The magnitude and quality of the secondary antibody response correlated with the abundance of these neutrophils, monocytes, and dendritic cells that were modified phenotypically and enriched prior to revaccination at 2 months, but not 2 weeks. These late phenotypic modifications were associated with an enhanced ex vivo cytokine production (including IL-12/23 and IL-1β) by PBMCs short after the second immunization, linking phenotype and functions. This integrated analysis reveals a deep impact of the timing between immunizations, and highlights the importance of early but also late innate responses involving phenotypical changes, in shaping humoral immunity.

## Introduction

Vaccination is one of the most important achievements made in the field of public health^1,2^. However, many vaccine-induced mechanisms are still unknown, limiting the design of vaccine immunogens and strategies, in particular for diseases such as AIDS, tuberculosis and malaria. Most vaccines require multiple injections to achieve a significant level of population (herd immunity) and individual immune protection^3–5^. However, the optimization of vaccine responses certainly requires a better understanding of how the time-interval between immunizations affects the molecular and cellular mechanisms inducing immune memory.

The early effector innate response initially triggers and shapes acquired immunity. Indeed, several systems vaccinology studies have reported the potential of innate immunity to predict long-term antibody (Ab) and T-cell responses^6^. Abs are the primary correlate of protection for most licensed vaccines^7^. It is thus critical to determine how innate immunity is induced by each immunization and how it associates with humoral immunity.

The modified vaccinia virus Ankara (MVA), a highly attenuated third-generation vaccinia-based smallpox vaccine, is a relevant vaccine model, as it induces both strong humoral and cellular immunity^8^. In contrast to vaccinia virus (VACV), from which it is derived and which provides life-long protection after a single administration, preclinical studies have shown that MVA requires a booster immunization to induce protective immunity^8^. Moreover, MVA is currently used as a vector to develop new recombinant vaccines against several diseases, including AIDS, tuberculosis, and malaria^9–11^. To refine its use, we need to increase our knowledge of its mode of action.

Non-human primates (NHP) are a well-recognized animal model for human vaccines research, given their close phylogenetic proximity to humans and their similar immune responses to several vaccines, including MVA^12,13^. We previously used cynomolgus macaques and MVA to show that two homologous subcutaneous injections 2 months apart induced a long-lasting specific antibody (Ab) response^14^. We also reported late changes in the phenotype of innate myeloid cells in the blood, including neutrophils, monocytes, classical dendritic cells (cDCs)^15^, and NK cells^16^, which occurred between 2 weeks and 2 months after the first immunization. However, systemic inflammation had long since resolved, with CRP and inflammatory cytokine concentrations and leukocyte counts back to baseline from 1-week post-first immunization. More precisely, these late phenotypic modifications of innate myeloid cells were characterized by the increased expression of several markers involved in signal transduction, antigen presentation, sensing, binding of immune complexes and complement, inflammation, and migration^15^. Consequently, the innate myeloid response to the second MVA exposure 2 months following the first injection involved phenotypically more activated/mature innate cells than the prime. We demonstrate here that shortening to 2 weeks the time-interval between MVA injections resulted in an impaired secondary Ab responses, and similar innate responses to both immunizations.

## Results

### Humoral responses induced by a second immunization 2 weeks or 2 months after the first immunization

**Fig. 1:**
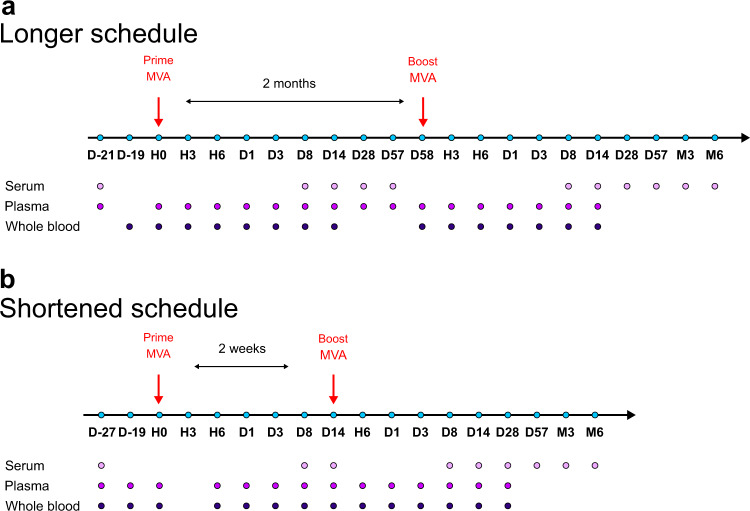
Overview of the experimental design. Five adult cynomolgus macaques were immunized twice subcutaneously with MVA HIV-B at a dose of 4 × 10^8^ PFU (**a**) 2 months apart (longer vaccine schedule)^14,15^ or (**b**) 2 weeks apart (shortened vaccine schedule). Blood samples were collected longitudinally at the indicated timepoints: hours (H), days (D), and months (M) post-prime (PP), and post-boost (PB). Immune responses were followed over time and compared.^14,15^. Animals from both schedules were immunized by the same route, at the same dose, with the same batch of vaccine. Only the vaccine schedules and the animals differed between studies.

**Fig. 2:**
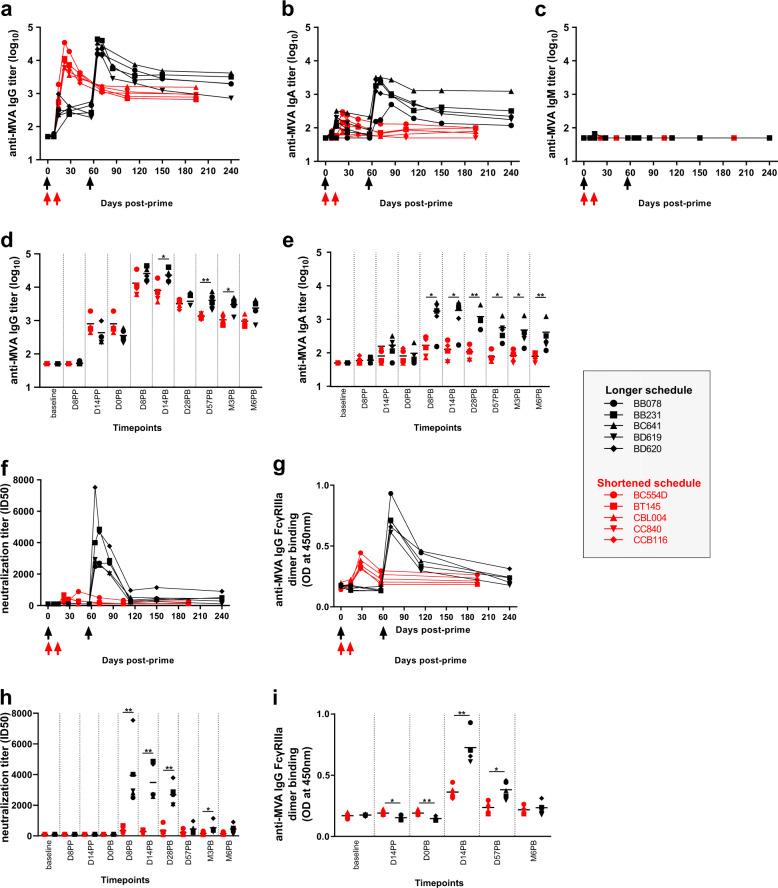
Serum MVA-specific antibody responses induced by a shortened and a longer vaccine schedule. MVA-specific (**a)** IgG, (**b)** IgA, and (**c)** IgM titers were measured by direct enzyme-linked immunosorbent assay (ELISA) in macaque serum over time. Comparisons of MVA-specific (**d)** IgG and (**e)** IgA titers were performed at the indicated timepoints between studies by aligning both vaccine schedules with respect to the date of the second immunization (2 weeks or 2 months after the first immunization). **f** MVA-nAb titers were quantified in serum using a single-cycle infection assay at the indicated timepoints. **g** The binding of serum MVA-specific IgG to FcγRIIIa was measured by ELISA using serum diluted as 1:500 and expressed as optical density (OD). Comparisons of (**h)** MVA-nAb titers and **i** binding of MVA-specific IgG to FcγRIIIa were performed at the indicated timepoints between studies. Immunizations are indicated by arrows. Colors indicate the vaccine regimen: first and second immunization 2 weeks (red), or 2 months (black) later. Individual titers or ODs are shown. Comparisons were performed using permutation tests (*p < 0.05, **p < 0.01).

Two groups of five cynomolgus macaques were immunized subcutaneously with a recombinant MVA HIV-B vaccine following two homologous injections, referred by convention as prime and boost. Two time-intervals between immunizations were tested: 2 months (longer vaccine schedule) or 2 weeks (shortened vaccine schedule) (Fig. 1). We first analyzed the impact of the timing of immunizations on the MVA-specific Ab response (Fig. 2).

Following the first MVA injection, we detected anti-MVA IgG and IgA responses in serum in both vaccine schedules (Fig. 2a, b) but no IgM response, except for macaque BB231 at D8PP and at a very low titer (Fig. 2c). Low concentration and affinity might have prevented IgM detection using these particular enzyme-linked immunosorbent assay (ELISA) settings. More precisely, all ten animals mounted an MVA-specific primary IgG response, which did not differ up to 2 weeks post first immunization, as expected (Fig. 2a, d). At the time of the second immunization (corresponding to 14 days or 58 days after the first immunization for the shortened and longer vaccine schedule respectively), the anti-MVA IgG titers were not statistically different (*p* = 0.76 using a permutation test). Both the short and long regimes resulted in a large increase in MVA-specific IgG titers. The secondary IgG response following the second MVA injection was statistically significantly higher in the 2 months interval group, at the peak response (week 2 post-second injection) and long after (6 months after the second immunization) (Fig. 2a, d), suggesting that the vaccine schedule impacts the recall and memory responses. MVA priming also led to a detectable MVA-specific IgA primary response, but only in some animals (four in the longer vaccine schedule and two in the shortened vaccine schedule) (Fig. 2b, e). This primary IgA response was lower than the primary IgG response (Fig. 2a, b), and comparable between the two groups up to 2 weeks post-MVA prime, as expected (Fig. 2d, e). At the time of the second immunization, 2 weeks or 2 months later, the anti-MVA IgA titers were comparable (*p* = 0.80 using a permutation test). However, the outcome of the second MVA injection differed highly between vaccine schedules and isotypes. There was a minor boost effect on the IgA response when the injection was performed at 2 weeks, as opposed to 2 months (fold change: 2.00 ± 0.54 for the shortened vaccine schedule and 19.26 ± 10.63 for the longer vaccine schedule). As a consequence, the MVA-specific secondary IgA response remained low (but detectable in all five animals) after the early second injection and lower than that after the late second injection, from peak to long-term response (Fig. 2b, e). In addition, MVA-specific Ab levels (IgG and IgA) decreased more rapidly after the early second injection (Fig. 2d, e and Supplementary Table 1). Finally, we further analyzed IgG subclasses and compared them between vaccine schedules. The specific IgG response was essentially polarized towards IgG1 (Supplementary Fig. 1a), with neither detectable IgG2 (Supplementary Fig. 1b) nor IgG3 (Supplementary Fig. 1c), regardless of vaccine regimen.

We next compared the capacity of MVA-specific Abs to neutralize MVA in vitro. Only secondary Abs, but not primary Abs, showed neutralizing activity (Fig. 2f). However, the secondary nAb response induced by the shortened vaccine schedule remained very low (with one animal with undetectable levels of nAb at D8) relative to that induced by the longer vaccine schedule, and was significantly lower at D8, D14, D28, and M3 following the second immunization (Fig. 2h). The serum neutralizing capacity rapidly decreased after 2 months, but remained detectable in all macaques of the 2 months interval group, with nAb titers of 516.4 ± 357.6 and 447.4 ± 298.5 at M3 and M6 post-boost, respectively.

We next investigated any differences in the ability of vaccine-induced IgG to interact with Fc receptors after binding to MVA. We used an ELISA based on recombinant dimeric FcγRIIIa, and described as a surrogate assay for influenza- and HIV-specific antibody-dependent cellular cytotoxicity (ADCC)^17,18^. Both the short and long regimens resulted in secondary anti-MVA IgGs capable of FcγRIIIa dimer binding (Fig. 2g), suggesting that they could cross-link FcγRIIIa (CD16) expressed by innate cells, such as NK cells, monocytes, and DCs, and activate them. We observed slight differences of primary responses between the two groups, but FcγRIIIa dimer binding was far stronger (higher optical density (OD)) after the second immunization at 2 months (Fig. 2i).

Overall, the time-interval between the first and second injection had little effect on secondary specific IgG titers, whereas it strongly modulated secondary IgA titers, serum neutralization activity, and binding of anti-MVA IgG to FcγRIIIa. It is likely that a second immunization at 2 weeks perturbs a lot the ongoing primary B-cell response and primary memory B-cell recall capacity, and thus the secondary humoral response. Innate immunity controls the memory B cell and antibody secreting cell development^19^. We previously reported that, in the longer vaccine schedule, innate myeloid responses strongly differed between prime and boost, with more activated/mature cells present prior to the second immunization at 2 months, and responding to it^15^. We thus next investigated whether the innate response to the second immunization at 2 weeks also differed from prime.

### Cytokine levels and leukocyte counts in response to MVA injections at 2 weeks interval

Plasma cytokines were analyzed after the first and second MVA immunization 2 weeks later (Supplementary Fig. 2 and Supplementary Table 2). Only a few cytokines (3 out of 24 soluble factors tested, IP-10, IL-6 and IL-1Ra) were significantly produced early and transiently in response to MVA immunizations (Supplementary Fig. 2a, b). We also analyzed the number of circulating leukocytes throughout the timecourse of vaccination (Supplementary Fig. 2c). There was a strong transient increase in cell number at H6 and D1 post immunizations (*p* < 0.01 for H6 and D1 post first immunization, and H6 post second immunization and *p* = 0.0159 for D1 post second immunization), but without any difference between the first and second MVA injection in terms of area under the curve (AUC), the leukocyte number being slightly lower 1 day after the second immunization than after the first one (*p* = 0.0317).

These results are comparable with those of the longer vaccine schedule^15^, with an immediate and rapidly resolved systemic inflammation similar after the first and second immunization. However, in our previous study, only the characterization of blood innate myeloid cells at a deeper resolution using mass cytometry revealed the induction of late phenotypic modifications of neutrophils, monocytes, and cDCs by the first immunization. This prompted us to assess the phenotype of innate myeloid cells in the shortened vaccine schedule.

### Phenotypic diversity of innate myeloid cells after two doses of MVA 2 weeks apart

**Fig. 3:**
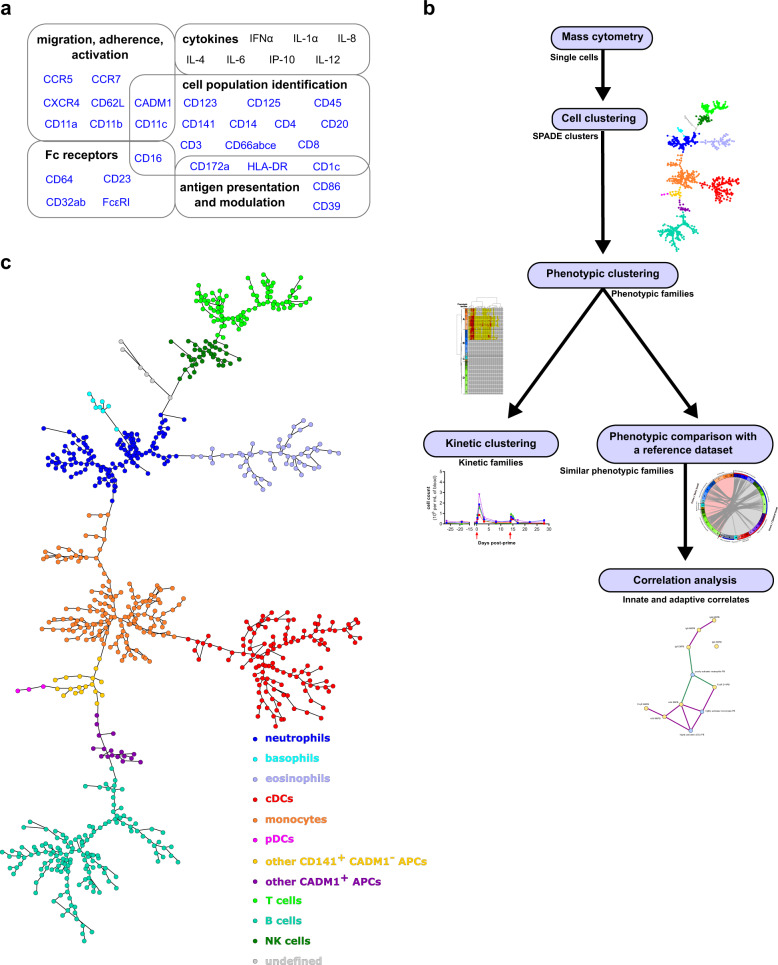
Bioinformatics pipeline used for the analysis of high-dimensional cytometry profiles. **a** A mass cytometry panel of 35 markers dedicated to the characterization of innate myeloid cells was used to stain fixed leukocytes obtained from cynomolgus macaque blood samples. Markers used for the SPADE clustering are indicated in blue. **b** The SPADE algorithm was used to identify groups of cells sharing similar phenotypes, called cell clusters. Phenotypic families, corresponding to groups of cell clusters with similar phenotypes, were defined for both the granulocyte and monocyte-DC compartments. Kinetic families, corresponding to phenotypic families sharing the same abundance profiles over time, were determined. Unsupervised phenotypic comparisons between the current shortened vaccine schedule study and a previously published longer vaccine schedule study^15^ were performed and correlations between innate myeloid responses and antibody responses were assessed. **c** The SPADE tree generated using all samples of the shortened vaccine schedule dataset and annotated based on the expression of 15 markers (Supplementary Fig. 3). Granulocyte clusters were defined as neutrophils (CD66^+^ CD125^−^), eosinophils (CD66^+^ CD125^+^), and basophils (CD66^−^ CD123^+^ HLA-DR^−^). Monocyte-DC clusters were defined as monocytes (CD14^+^ HLA-DR^+^), cDCs (CD14^−^ HLA-DR^+^ CD11c^+^ CD16^+^), inflammatory cDCs/nonclassical monocytes (CD14^+^ HLA-DR^+^ CD11c^+^ CD16^+^), pDCs (CD123^+^ HLA-DR^+^), other CADM1^+^ APCs (HLA-DR^+^ CD3^−^ CD8^−^ CD14^−^ CD11c^−^ CD16^−^ CD20^−^ CADM1^+^), and other CD141^+^ CADM1^−^ APCs (HLA-DR^+^ CD3^−^ CD8^−^ CD14^−^ CD11c^−^ CD16^−^ CD20^−^ CD141^+^ CADM1^−^). Lymphocyte clusters were defined as B cells (CD20^+^ HLA-DR^+^), T cells (CD3^+^), and NK cells (CD3^−^ CD8^+^). The undefined category corresponds to cell clusters that did not fit any of these phenotypes.

We stained whole blood with a 35-antibody panel detailed in Fig. 3a and Supplementary Table 3 and used an analysis pipeline consisting of three successive clustering steps (Fig. 3b). The SPADE algorithm was applied to identify leukocyte clusters sharing similar phenotypes within the entire dataset, as previously done in the longer vaccine schedule study^15,16^. The whole dataset was composed of fixed leukocytes from the five macaques immunized with MVA at 2 weeks interval and collected at 13 timepoints (Fig. 1a). The parametrization of the SPADE algorithm was optimized and we identified 800 clusters based on the following 28 clustering markers: CADM1, CCR5, CCR7, CD1c, CD3, CD4, CD8, CD11a, CD11b, CD11c, CD14, CD16, CD20, CD23, CD32, CD39, CD45, CD62L, CD64, CD66, CD86, CD123, CD125, CD141, CD172a, CXCR4, FcεRI, and HLA-DR.

Cell clusters were annotated on the resulting SPADE tree (Fig. 3c) based on the expression of CADM1, CD1c, CD3, CD4, CD8, CD11c, CD14, CD16, CD20, CD66, CD123, CD125, CD141, CD172a, and HLA-DR (Supplementary Fig. 3). We identified 192 clusters of granulocytes that included CD66^high^ CD125^−^ neutrophils, CD66^−^ HLA-DR^−^ CD123^+^ basophils, and CD66^mid^ CD125^+^ eosinophils. We found 322 clusters of monocytes-cDCs that included HLA-DR^+^ CD14^−^ CD11c^+^ CD16^+^ CD141^+^ cDCs (including CADM1^+^ cDC1s and CADM1^−^ cDC2s), HLA-DR^+^ CD14^+^ CD141^+^ monocytes (including HLA-DR^+^ CD14^+^ CD11c^+^ CD16^+^ nonclassical monocytes/inflammatory cDCs), HLA-DR^+^ CD123^+^ plasmacytoid DCs (pDCs), HLA-DR^+^ CD3^−^ CD8^−^ CD14^−^ CD11c^−^ CD16^−^ CD20^−^ CD141^+^ CADM1^−^ antigen-presenting cells (APCs), and HLA-DR^+^ CD3^−^ CD8^−^ CD14^−^ CD11c^−^ CD16^−^ CD20^−^ CD141^+/−^ CADM1^+^ APCs. Furthermore, we identified 280 cell clusters of lymphocytes that included CD3^+^ T cells, HLA-DR^+^ CD20^+^ B cells, and CD3^−^ CD8^+^ NK cells. Six clusters remained undefined (CD66^−^ CD125^−^ CD3^−^ CD4^−^ CD8^−^ HLA-DR^−^ CD20^−^ CD14^−^ CD11c^−^ CD123^−^ CD16^−^ CADM1). As our panel was solely dedicated to target innate myeloid cells, we next focused the analysis on the granulocyte and monocyte-DC compartments.

**Fig. 4:**
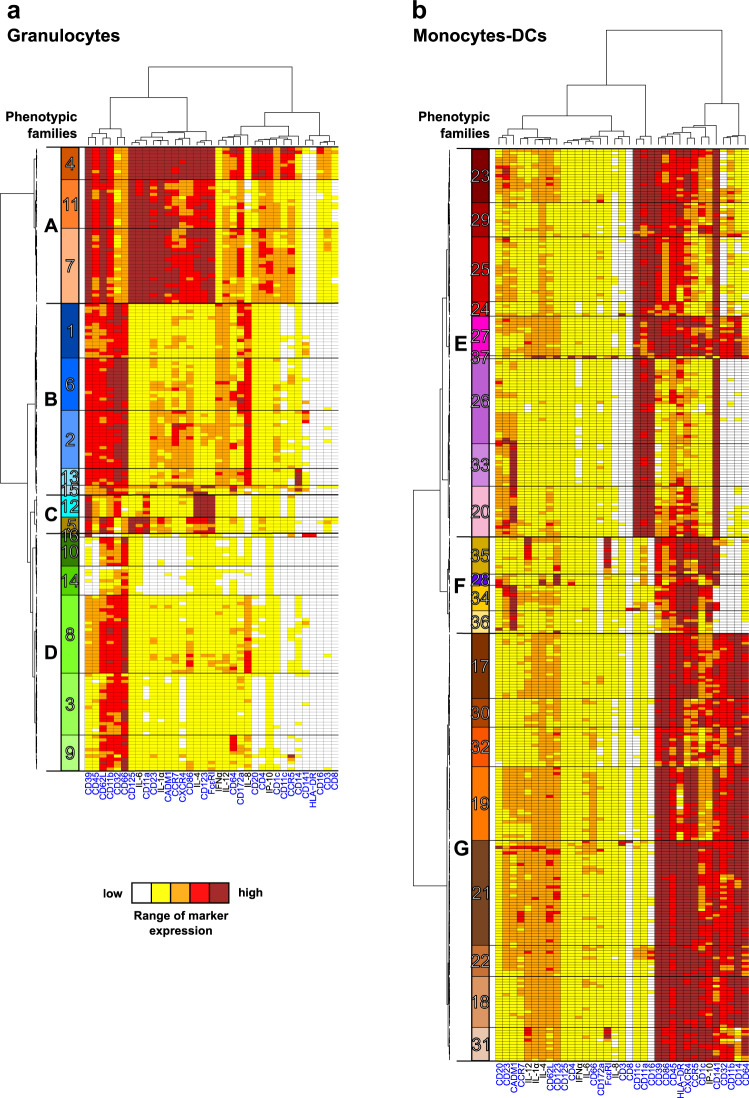
Phenotypic diversity of granulocytes and monocytes-DCs in the shortened vaccine schedule dataset. Categorical heatmaps showing relative marker expression for (**a)** granulocyte or (**b)** monocyte-DC clusters. Each row corresponds to a cell cluster, and each column corresponds to a cell marker. Phenotypic families, corresponding to groups of cell clusters sharing similar phenotypes, were delineated based on the cluster dendrogram. The 16 granulocyte phenotypic families (numbered from 1 to 16) and the 21 monocyte-DC phenotypic families (numbered from 17 to 37) are indicated in different colors. Black frames labeled with capital letters indicate superfamilies of phenotypic families. SPADE clustering markers are indicated in blue. Interactive heatmaps are available at http://data.idmitcenter.fr/MVA-innate-myeloid-early-boost/.

We displayed these cell cluster phenotypes on two categorical heatmaps of marker expression to decipher more easily the phenotype of innate myeloid cells, one for granulocytes and one for monocytes-DCs, after hierarchical clustering of the cell clusters and markers (Fig. 4). Based on the cell cluster dendrogram, we regrouped cell clusters sharing similar phenotypes into so-called phenotypic families and phenotypic families into super (phenotypic) families, and we annotated them.

In the granulocyte compartment (Fig. 4a), 16 distinct phenotypic families (randomly numbered from 1 to 16) were distinguished and further regrouped into four phenotypic superfamilies (named from A to D). Eosinophils (phenotypic families 4, 11, and 7 composing the superfamily A) clearly segregated apart from the rest of the granulocytes. All eosinophils displayed high expression of CD39, CD45, CD62L, CD11b, CD125, CD23, IL-1α, CADM1, CCR7, CXCR4, CD86, IL-4, CD123, and FceRI, as well as mid-level expression of CD66, and CD32. However, they expressed IL-12, CD64, CD172a, CD20, CD4, IP-10, CD11c, and CCR5 at different levels (high, low, and mid for phenotypic families 4, 11, and 7, respectively). This surprising signature suggests a highly activated phenotype. However, caution should be taken in the interpretation of these phenotypes, as eosinophils could potentially bind to lanthanides, despite the use of heparin during staining^20^.

Neutrophils (phenotypic superfamilies B and D) were largely represented among granulocytes as expected, and they displayed some phenotypic heterogeneity. We found CD39^high^ CD45^high^ IL-8^high^ neutrophils (phenotypic families 1, 6, 2, 13, and 15 composing the superfamily B), as well as CD39^low/mid^ CD45^mid^ IL-8^mid^ neutrophils, which may be less active/mature (phenotypic families 16, 10, 14, 8, 3, and 9 from the superfamily D). The various patterns of expression of CCR7, CXCR4, CD86, CD123, and FcεRI by neutrophils belonging to the phenotypic family 15 from the superfamily B, might be related to their immaturity^21^. We also identified CD14 expressing neutrophils (phenotypic family 13, superfamily B). This feature has been previously reported in some neutrophils, without its function being clearly defined^22^. We detected neutrophils expressing CD172a, an inhibitor of phagocytosis in macrophages, also involved in the regulation of neutrophil transmigration^23^ (phenotypic families 1 and 6 from superfamily B). Finally, the single CD66^low^ HLA-DR^+^ cell cluster composing the phenotypic family 16, superfamily D may actually correspond to APCs.

Finally, basophils comprised one phenotypic family (phenotypic family 12 from the superfamily C). They ended up clustered together with CD23^low^ IL-1α^low^ CADM1^low^ CCR7^low^ CXCR4^low^ CD86^low^ CD66^mid^ CD123^high^ CD125^high^ eosinophils (phenotypic family 5, superfamily C).

Among the monocyte-DC compartment (Fig. 4b), we identified 21 phenotypic families (17−37) and grouped them into three distinct superfamilies. cDCs (phenotypic families 23, 29, 25, 24, 27, 37, 26, 33, and 20) and nonclassical monocytes/inflammatory cDCs (phenotypic family 27) grouped together into the phenotypic superfamily E. CADM1^high^ CD39^low/mid^ CD86^low/mid^ CD45^low/mid^ HLA-DR^low/mid^ CXCR4^low/mid^ CCR5^low/mid^ CD1c^low/mid^ IP-10^low/mid^ cDCs (phenotypic families 20 and 33) may be considered to be cDC1s, whereas CADM1^low^ cDCs (phenotypic families 26, 24, 25, 29, and 23), also displaying varying levels of CD39, CD86, CD45, HLA-DR, CXCR4, CCR5, CD1c, and IP-10, may be considered to be cDC2s. These results must be regarded however with caution, as the signal for CD172a was lower in cDCs than granulocytes, which resulted in most cDCs appearing in very low (white) or low (yellow) categories, preventing us to firmly use it as cDC2 marker. Phenotypic family 37 contained one single cluster, with a patchwork phenotype, including CD66 expression. It may actually correspond to granulocytes.

Monocytes, which composed the superfamily G (phenotypic families 17, 30, 32, 19, 21, 22, 18, and 31), expressed high levels of CD39, CD86, CD45, CD11b, and CD64. They were positive (but with varying levels of expression) for HLA-DR, CXCR4, CCR5, CD1c, IP-10, CD141, CD32, and CD14.

Other APCs were found in the superfamily F (phenotypic families 35, 28, 34, and 36). They comprised HLA-DR^+^ CD14^−^ CD11c^−^ CD16^−^ CD20^−^ CD141^+/−^ CADM1^+^ APCs segregated into CD1c^low^ (phenotypic family 36) and CD1c^high^ (phenotypic family 34) APCs, pDCs (phenotypic family 28), and HLA-DR^+^ CD14^−^ CD11c^−^ CD16^−^ CD20^−^ CD141^+^ CADM1^−^ IL-12^high^ CD172a^high^ IP-10^high^ APCs (phenotypic family 35).

### Similar innate myeloid cell responses after a first and a second MVA injection shortly afterwards

**Fig. 5:**
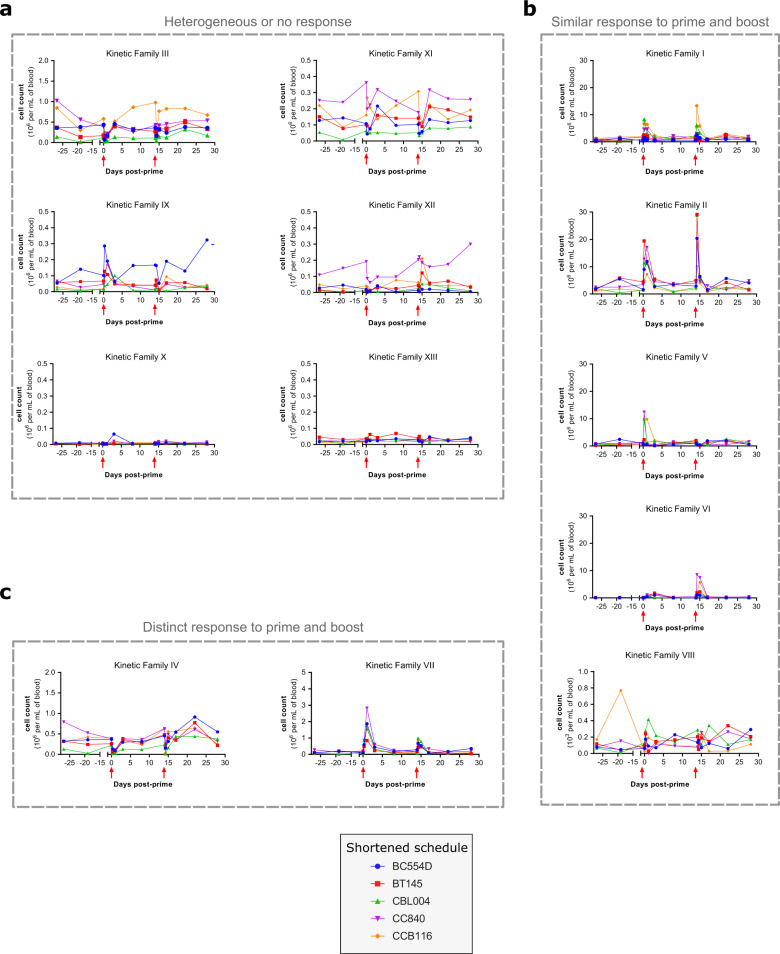
Kinetic profiles of innate myeloid cells after immunizations 2 weeks apart. The abundance profiles over time of 13 kinetic families (numbered I to XIII) are displayed. Kinetic families are composed of granulocyte (Supplementary Fig. 4) and monocyte-DC (Supplementary Fig. 5) phenotypic families sharing similar dynamics. Kinetic families were further regrouped based on their kinetic patterns (Supplementary Table 5) as indicated in gray, (**a**) heterogeneous or no response, (**b**) similar response to prime and boost, (**c**) distinct response to prime and boost. The immunizations are indicated by red arrows. The scale of the *Y*-axis is specific to each kinetic family.

Beyond the phenotypic diversity within the dataset, we were primarily interested in the behavior of these cells over time after the prime and second boost 2 weeks later. We thus grouped the granulocyte (Supplementary Fig. 4) and monocyte/DC (Supplementary Fig. 5) phenotypic families sharing the same kinetics into so-called kinetic families (randomly numbered from I to XIII in roman numerals) (Fig. 5) to analyze the dynamics of the innate immune responses to MVA short interval injections (Supplementary Table 4).

Among these 13 kinetic families, six (families III, IX, X, XI, XII, and XIII) showed no response or heterogeneous responses to MVA injections, because of high variability between animals at baseline or post immunization (no statistically significant *p* value for comparison of cell counts with baseline) (Fig. 5a and Supplementary Table 5).

In contrast, five kinetic families (families I, II, V, VI, and VIII) responded similarly to each MVA administration, showing no statistical difference for the comparison of the first and second injection AUCs and at least one statistical difference for the comparison of cell counts at a given timepoint with baseline (Fig. 5b and Supplementary Table 5). Kinetic families I, II, and VI underwent a rapid and transient increase of cell counts after each MVA immunization. They were composed of more-or-less activated neutrophils and monocytes (Supplementary Table 4). Kinetic family V was characterized by a nonstatistically significant increase of cell counts at H6 post-prime (*p* = 0.0556), followed by a significant decrease 1 day following the second immunization. It contained neutrophils, including CD14^+^ neutrophils, and monocytes, with a more-or-less activated phenotype (varying notably by CD1c expression). Finally, kinetic family VIII, which corresponded to rare CD141^+^ HLA-DR^+^ CD66^low^ cells annotated as neutrophils, but which may correspond to peculiar APCs, showed an increase in cell counts very early after the first immunization and then a decrease 1 day following the second immunization (Fig. 5b).

Eventually, two kinetic families (families IV and VII) reacted to both immunizations, but differently (*p* < 0.05 for the comparison of the first and second injection AUCs) (Fig. 5c and Supplementary Table 5). Kinetic family IV, comprising CD123^+^ eosinophils and basophils, showed a decrease after both immunizations, as well as an increase 1 week after the second immunization. Kinetic family VII, composed of monocytes (including HLA-DR^low^ monocytes), pDCs, and inflammatory cDCs/nonclassical monocytes, showed an increase after each immunization, but with an attenuated magnitude in the response to the second immunization relative to prime.

The representation of the phenotypic family composition over time with pie charts also supports that there was no major difference between the response to prime and early boost (Supplementary Fig. 6). We eventually used a multidimensional scaling representation to visualize in a 2D plot the heterogeneity and similarity between samples with respect to subphenotype composition, while better accounting for the magnitude of the responses to MVA injections, and not only for their quality (Supplementary Fig. 7a). Most timepoints overlapped, with the exception of H6 and D1 post first immunization, and H6 post second immunization. D1 post second immunization colocalized with the other samples, suggesting that the response to the second injection at 2 weeks was comparable to that of the first injection, it was only resolved faster. These results highly contrasted with the longer vaccine schedule study^15^. Indeed, in that study, H6 and D1 post first immunization, as well as H6 post second immunization clearly segregated apart from the other samples, but also from one another, strongly indicating that the innate myeloid response after a boost at 2 months, but not at 2 weeks, differ from the response to the prime.

Two kinetic families still behaved differently between immunizations at 2 weeks interval. We thus evaluated whether it was sufficient to discriminate between responses to the first and second immunization 2 weeks later using a multivariate LASSO approach. The cross-validation could only build a model with a very high error rate for class prediction. The minimal mean square error (MSE) was 0.76, which was approximately four times less accurate than the model built for the longer vaccine schedule (MSE = 0.24) (Supplementary Fig. 7b). This analysis firmly establishes that the response to prime and early second injection cannot be distinguished from each other, contrary to the response to prime and delayed second injection.

### Missing activated/mature innate myeloid cells in the shortened vaccine schedule dataset

**Fig. 6:**
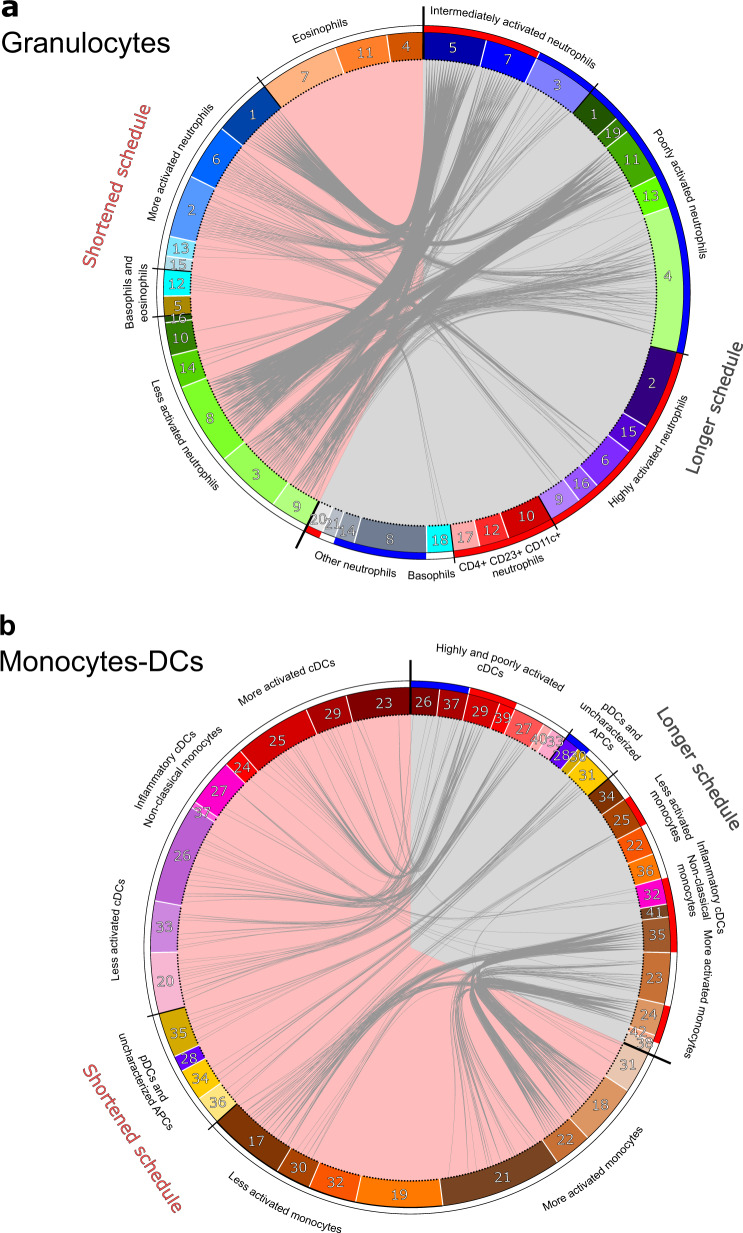
Comparisons of cell cluster phenotypes identified in the two vaccine schedules. The phenotype of each cluster of (**a)** granulocytes and (**b)** monocytes-DCs of the current shortened vaccine schedule study (left, pink background) was compared to that of clusters identified in the previous longer vaccine schedule study (right, gray background)^15^. The similarity between clusters was computed as the Manhattan distance, calculated for all the categories of expression of the 27 markers shared in both experiments. Clusters were considered to be similar if the in-between distance was strictly below 10 and they were linked together in the circular graph. For the shortened vaccine schedule dataset, the color code for the phenotypic families is identical for the circular graph (Fig. 6) and heatmaps (Fig. 4), whereas it has been modified for the previously published longer vaccine schedule^15^ to match identical annotations in both datasets. Phenotypic family annotations are displayed. For the longer vaccine schedule dataset, phenotypic families that were selected using the LASSO-LDA approach to distinguish prime and boost responses^15^ are represented with a blue (prime) or red (boost) color band.

We then formally associated the cellular phenotypes identified in the shortened and longer vaccine schedule datasets to identify those present in both datasets and those retrieved in only one. The comparison was challenging because we used different mass cytometers, different Ab panels and Ab batches, and different staining protocols. We compared the categorical heatmaps created for each study (Fig. 3 and Palgen et al.^15^) and used the Manhattan distance as a read-out of the similarity between cell cluster phenotypes (Fig. 6).

All neutrophils phenotypic families from the shortened vaccine schedule study (superfamily D and B) associated with poorly (superfamily C) to moderately (superfamily B) activated neutrophils from the longer vaccine schedule study. We observed a few rare associations with the highly activated neutrophils (superfamily A) and none with CD4^+^ CD23^+^ CD11c^+^ neutrophils (superfamily D), present only in the longer vaccine schedule study (Fig. 6a). Basophils from each study (phenotypic family 12 for the early second immunization and phenotypic family 18 for the delayed second immunization study) were associated with each other. However, eosinophils did not match. This was expected because eosinophils were characterized only in the shortened vaccine schedule (superfamily A and phenotypic family 5), whereas they had been removed from the analysis of the longer vaccine schedule study due to nonspecific staining^20^. We observed an additional discordance, consisting of the lack of association of CD14^+^ neutrophils between the accelerated vaccine schedule dataset and the delayed vaccine schedule dataset (phenotypic family 13 in both regimens). CD14^+^ neutrophils from the prime/boost at 2 weeks dataset associated with intermediately activated neutrophils from the prime/boost at 2 months dataset instead (superfamily B). This may have been related to the differential expression of other markers (CD66 and CD11b) by CD14^+^ neutrophils from both studies.

Most cDCs and inflammatory cDCs/nonclassical monocytes (superfamily E), as well as uncharacterized APCs (superfamily F), from the shortened vaccine schedule study matched their counterparts in the longer vaccine schedule boost study (Fig. 6b). Surprisingly, this was not the case for pDCs (phenotypic family 28 in both schedules), which were not associated between the two datasets, likely due to the higher expression of CD20 by pDCs in the shortened vaccine schedule study than in the longer vaccine schedule study. Most monocytes from the shortened vaccine schedule dataset (superfamily G) also associated with monocytes from the longer vaccine schedule dataset (superfamily H), except phenotypic families 19 and 32 from the accelerated vaccine regimen dataset. They corresponded to HLA-DR^low^ and CCR5^mid^ CXCR4^mid^ monocytes, respectively, and were not linked to phenotypic families 22 or 36 (HLA-DR^low^ monocytes), or 25 (CCR5^mid^ CXCR4^mid^ monocytes) from the the delayed vaccine regimen dataset. This discrepancy may have been due to the differential expression of other markers (CD32, CD11b, CD11a, and CD14) between HLA-DR^low^ monocytes and CCR5^mid^ CXCR4^mid^ monocytes in the two studies.

Although most phenotypic families of the shortened vaccine schedule study associated with their counterparts of the longer vaccine schedule study, the inverse was not true. Indeed, most phenotypic families from the injections 2 months apart dataset previously classified as characteristic of the response to boost by LDA (which discriminated responses to the first and second immunization 2 months later) were absent from the shortened vaccine schedule dataset, such as highly activated neutrophils and CD4^+^ CD23^+^ CD11c^+^ neutrophils^15^. This was more pronounced for granulocytes than monocytes and DCs, with seven nonassociated phenotypic families among the 11 that defined the boost at 2 months signature in the LDA model for granulocytes versus one among six for monocytes-DCs.

Overall, these results strengthen the body of evidence demonstrating that early second immunization did not result in an innate myeloid cell immune response different from prime, in contrast to a later second immunization, which involved more mature/activated cells.

### Correlation between secondary humoral and innate myeloid cell responses

**Fig. 7:**
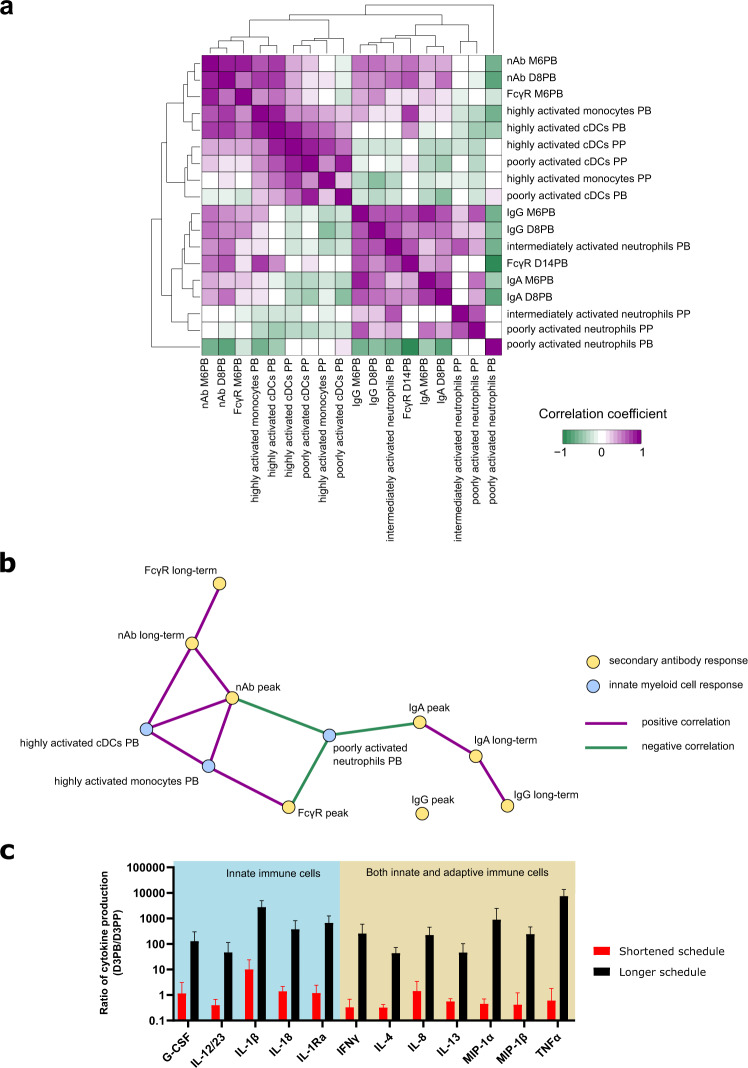
Correlation between innate myeloid subphenotypes and humoral response and link between phenotype and function. **a**, **b** The correlations between the peak (8 or 14 days post-boost, D8 or D14PB) and long-term persistence (6 months post-boost, M6PB) of the secondary antibody response and the innate myeloid cell response were assessed. **a** All associations are displayed after hierarchical clustering of the correlation coefficients with complete linkage. **b** All Ab response variables used for the correlation analysis are represented (in yellow), whereas only the innate myeloid response variables that correlated (|*R*| > 0.7, positive correlations in plum and negative correlations in green) with at least one Ab response variable (in blue) are displayed on the correlation graph. **c** The ex vivo cytokine production of unstimulated PBMCs was assessed 3 days post-prime (D3PP) and post-boost (D3PB) (both vaccine schedules). For each of the indicated cytokines, the ratio of cytokine level at D3PB to the level at D3PP is represented as the mean ± standard deviation for the five animals. The main immune cell producers are also indicated. Only cytokines with a statistically significant different ratio between the two schedules, with at least one ratio >10, are represented. Significance was considered to be *p* ≤ 0.01 by the Mann−Whitney−Wilcoxon test.

Given the striking differences in secondary humoral and innate myeloid cell responses observed between vaccine schedules, we investigated to what degree they related to each other. We thus analyzed the correlations between the peak of the secondary Ab response and its long-term persistence and the early innate responses to immunizations. More precisely, we associated four Ab variables (MVA-specific IgG, IgA, and nAb titers, and MVA-specific IgG binding to FcγRIIIa) at two timepoints (at the peak, 8 or 14 days after the second immunization, and in the long term, 6 months after the second immunization) with the abundance of five innate myeloid-cell populations (poorly activated neutrophils, intermediately activated neutrophils, highly activated monocytes, poorly activated cDCs, and highly activated cDCs) post-prime and -boost (Fig. 7a). We did not use highly activated neutrophils or poorly activated monocytes for this multivariate correlation analysis, because they were not present in both datasets.

We found associations between monocytes and cDCs and early and late nAb titers and late IgG binding to FcγRIIIa, as well as between neutrophils and early and late IgG and IgA titers and early IgG binding to FcγRIIIa (Fig. 7a). Post-boost poorly activated neutrophil counts inversely correlated with peak IgA and nAb titers and IgG/FcγRIIIa binding, whereas post-boost highly activated monocytes and cDCs positively correlated with peak nAb titers and IgG/FcγRIIIa binding and peak and long-term nAb titers, respectively (Fig. 7b). In contrast, intermediately activated neutrophils did not correlate with any variable, possibly because they responded to both first and second immunization. In addition, post-prime innate responses showed only low correlations (|*R*| < 0.7) and were not considered to be significant.

We conclude that a longer interval between immunizations made it possible to take advantage of the presence of prime-induced phenotypically modified innate myeloid cells prior to revaccination and ensure good recall of the Ab response. We next investigated how the phenotypic differences between cells responding to MVA injections translate into functions.

### Enhanced ex vivo inflammatory cytokine production by PBMCs after the second vaccine injection at 2 months but not at 2 weeks

PBMCs were isolated after MVA in vivo stimulation, 3 days after the first and second injection (both vaccine schedules), and cytokine production was assessed without any ex vivo re-stimulation after overnight culture. The production of many cytokines (G-SCF, IL-12/23, IL-1β, IL-18, IL-1Ra, IFNγ, IL-4, IL-8, IL-13, MIP-1α, MIP-1β, and TNFα) was much higher after the second immunization at 2 months than prime (ratios ranging from 44 to more than 7000). This was not true for the second immunization at 2 weeks (ratios about 1), with the notable exception of IL-1β, of which the production was also higher after early boost than prime (ratio of 10). Many of these cytokines (IFNγ, IL-4, IL-8, IL-13, MIP-1α, MIP-1β, and TNFα) can be secreted by both innate and adaptive cells^24,25^, but others (G-SCF, IL-12/23, IL-1β, IL-18, and IL-1Ra) suggest an innate-origin of the producing cells, including innate myeloid and lymphoid cells. We were unable to consider the contribution of granulocytes to this enhanced cytokine response because PBMCs, and not whole blood or leukocytes, were used for this functional assay. In any case, these results clearly demonstrate a greater innate effector response to revaccination at 2 months than to vaccination or to revaccination at 2 weeks. However, this type of functional analysis cannot fully resolve whether the increased cytokine response corresponded to the enhanced intrinsic responsiveness of innate cells to MVA re-encounter (reminiscent of innate immune training), and/or to their enhanced activation and licensing by immune complexes and restimulated primary T cells^26^, of which their functions depend on time after initial antigen exposure^27^. Nonetheless, as previously shown, IL-12 expression was augmented in monocytes and cDCs induced by prime, present prior to a second immunization at 2 months, responding to it, and characteristic of it based on the LDA classification^15^. Its increased secretion by PBMCs collected soon after a second immunization at 2 months, but not at 2 weeks, compared to prime (Fig. 7c) links the late prime-induced phenotypic modifications of innate cells and their resulting innate functions at boost. This result favors the hypothesis of the involvement, at least in part, of trained immunity in the enhanced innate response to revaccination after 2 months.

## Discussion

We evidence that the timing of immunizations is important for harnessing prime-induced activated/mature innate myeloid cells to respond to vaccine re-encounter and efficiently boost the Ab response for longer-lasting protective immunity. Indeed, by comparing macaques immunized with MVA according to two different schedules, shortened or longer, and phenotyping their blood innate myeloid cells over time by mass cytometry, we reported that, conversely to a second immunization at 2 months, a second immunization at 2 weeks did not induce an innate myeloid response different from that of prime. Indeed, none of the statistical models we created was able to fully discriminate between the responses to prime and early second injection, in contrast to prime and later second injection. A correlation analysis between the innate myeloid and secondary Ab responses showed that poorly activated neutrophils responding to boost inversely correlated with peak secondary IgA and nAb titers and IgG/FcγRIIIa binding, whereas highly activated monocytes and cDCs positively correlated with peak nAb titers and IgG/FcγRIIIa binding and peak and long-term nAb titers, respectively.

Through the use of mass cytometry, we were able to deeply phenotype innate myeloid cells from the blood of immunized macaques. We shed light on new cell populations, such as CADM1^+^ APCs and FcεRI^+^ CD141^+^ APCs. However, CD172a expression by cDCs was unexpectedly low in our settings relative to that reported in the literature^28^. This may have been caused by the sample fixation prior to staining. Admittedly, it remained challenging to precisely delineate and annotate macaque monocytes and cDCs subsets, based solely on high-dimensional cytometry data, without scRNA-seq data, as recently done for human mononuclear phagocytes^29^. We also unveil several new features of neutrophils, such as their wide expression of CD39. We also found a population of CD123^+^ eosinophils. IL-3 was previously shown to regulate eosinophil function, likely through CD123 binding^30,31^. Strikingly, these CD123^+^ eosinophils were among the very few cell populations that were more abundant after early boost than prime. Eosinophils were not however analyzed in the longer vaccine schedule, as the staining protocol using heparin to limit interactions between eosinophil granule contents and lanthanides was not used^15,20^.

It may be tempting to shorten the vaccine schedule in face of a new emerging infectious disease or outbreak, and hence ensure faster protection of the population at risk, but an accelerated vaccination schedule appears to be detrimental for humoral immunity. Such a large impact of the time-interval between first and second immunizations on the intensity and quality of the specific Ab response is concordant with clinical studies showing that a delay between injections shorter than 3 weeks impaired the ability of individuals to develop protective immunity against smallpox after MVA immunizations^32,33^. As for recombinant MVA vaccine vectors, in a recent clinical trial evaluating an MVA-based influenza A H5N1 vaccine, a boosting immunization 1 year after the prime was shown to elicit higher neutralizing antibody titers compared to a boost at 1 month against influenza, although the level of sero-conversion was similar in both groups^34^. Strikingly, the ADCC activity of the anti-H5 antibodies were strongly increased in the late (1 year) boost schedule, but not in the early (1 month) boost schedule^35^.

Undoubtedly, the dynamics of the memory differentiation of specific primary B and T cells play a key role in determining the optimal interval between the first and second immunization^36^. Our abbreviated schedule likely prevented the germinal center reaction to fully mature after prime and generate the somatic hypermutations required to increase BCR affinity in a way to produce nAb. In addition, it did not allow for a period of quiescence, as discussed recently regarding an HIV envelope trimer-based candidate vaccine evaluated in NHP^37^. However, our data highlight an additional rationale for delaying the timing of a second immunization: to leave enough time for the phenotypically modified innate myeloid cells to develop in response to prime to optimally re-stimulate primary memory B at the time of boost. Moreover, we reveal that not only the early effector innate responses, as repeatedly reported^38,39^, but also late innate responses shape adaptive immunity. Although innate immunity has not been assessed in the MVA-based H5N1 influenza virus clinical trial^34,35^, we hypothesize that innate cells could also play a tremendous role in the boost effect of the late second immunization on the Ab response, as suggested by our study, and that the contribution of foreign antigens expressed by the recombinant vaccine is likely minor compared to the one of the nature of the vaccine vector.

Though formal protection against poxvirus challenge was not assessed in this study, the level of neutralizing antibody observed in the longer, but in not the shortened vaccine schedule, were higher than the one of VIGIV (a pool of immunoglobulins from humans who received recent booster immunizations with VACV), which protected rhesus macaques against monkeypox after passive transfer^40^. This suggests that the phenotypic modifications we observed on the innate myeloid cells are not only predictors of the quality and quantity of the antibody response, as shown by the correlations with neutralizing Ab and FcγR binding (Fig. 7a, b), but also very likely indirect predictors of the protection mediated by antibodies. Whether the MVA-induced phenotypic modifications of innate myeloid cells would also translate into increased antiviral functions providing direct protection against poxviruses, and even heterologous pathogens, remains to be tested.

The question arises as to whether the phenotypically modified innate myeloid cells induced by MVA prime and present 2 months later, but not to 2 weeks, correspond to bona fide trained cells. Innate memory cells are commonly defined by their enhanced in vitro functional responses, usually cytokine secretion, to unrelated stimuli. This has currently been demonstrated for monocytes and NK cells. Although not highly emphasized in the literature, one feature of trained monocytes is their upregulated expression of CD14, CD11b, and TLR4 after BCG vaccination^41,42^. Although formal evidence of training is absent, macaque neutrophils were recently reported to express higher levels of CD11b, TNF-a, IL-21, MPO, and ROS 2 weeks after a second mucosal immunization with a replication-competent adenovirus-SIV recombinant than before any immunization^43^. After physiological microbial exposure of laboratory mice by cohousing with pet store mice, TLR4^+^ monocyte, TLR2^+^ monocyte, and TLR2^+^ neutrophil counts increased^44^. Additionally, training of DCs was recently reported. Murine DCs produced more cytokines in response to *Cryptococcus neoformans* and *Staphylococcus aureus* 70 days after pulmonary immunization with a recombinant *C. neoformans* strain expressing IFNγ, in contrast to naïve mice^45^. Two months, but not 2 weeks, after MVA prime and prior to MVA revaccination, we previously showed that blood monocytes, neutrophils, and cDCs were phenotypically “defense-ready”. They expressed higher levels of several markers, such as molecules involved in signal transduction (CD45), antigen presentation (HLA-DR), sensing (CD14), binding of immune complexes (CD16, CD32), and complement (CD11b, CD11c), inflammation (IL-10, IP-10, IL-12, IL-8), and migration (CXCR4, CCR5)^15^. We also found that PBMCs collected early after the second immunization at 2 months (i.e. 3 days after in vivo re-stimulation with MVA) produced more inflammatory cytokines than those collected after the first immunization or second immunization at 2 weeks. At this stage, we cannot rule out the contribution of primary memory B and T cells and specific Abs in the increased production of innate cytokines, such as IL-12, by innate cells. However, we can associate the modified phenotypes induced by prime and pre-existing to the delayed second immunization (and thus independent of the re-stimulation of primary memory B and T cells by MVA and the presence of MVA/Ab immune complexes, except if, somehow and unexpectedly, MVA persisted and blipped) with an improved innate response to revaccination. This association strongly suggests that MVA, like BCG, enhanced the intrinsic responsiveness of neutrophils, monocytes, and cDCs. Admittedly, additional functional and mechanistic experiments are required to obtain a definitive conclusion.

Observational studies have previously shown that Vaccinia virus smallpox vaccine provides nonspecific protection against overall mortality^46^. It was recently reported, using human primary monocytes stimulated in vitro with VACV or MVA for 1 day and challenged a week later with unrelated stimuli, that monocytes treated with VACV produced more proinflammatory cytokines in response to heterologous pathogen-associated molecular patterns, whereas monocytes previously stimulated with MVA produced less. The authors concluded that VACV induced trained immunity, but, on the contrary, MVA induced innate immune tolerance^47^. They acknowledged the limits of their study, which was not comparative, since neither the physical dose nor the infectious dose of the viruses, VACV or MVA, were controlled or equal. This also suggests that either monocyte/macrophage training by MVA, if any, as suggested by our studies, requires the actions of other cell types to occur, for example effector CD8 T^+^ cells and the IFNγ they produce, as for alveolar macrophages after respiratory adenoviral infection^48^, or that training is performed not (only) at the differentiated innate cell level, but also/rather at the hematopoietic progenitor cell level. Thus, the in vitro model of training using monocytes would not fully recapitulate what happens in vivo. Indeed, training of hematopoietic stem cells was demonstrated in mice after intravenous immunization with BCG^49^ or after intraperitoneal injection of β-glucan^50^ and resulted in trained monocytes protecting against virulent *Mycobacterium tuberculosis* challenge or LPS challenge and chemotherapy-induced myelosuppression, respectively. This mechanism of hematopoiesis reprogramming responsible for the training of innate cells is consistent with our observations that only waiting 2 weeks after MVA prime, in contrast to two months, was not sufficient to generate “better equipped” innate cells to circulate in the blood to be mobilized at boost. The more upstream the rewired progenitor is, the longer the required time of differentiation to generate a modified progeny. The time to recovery of normal blood neutrophil counts after myeloablative total body irradiation is approximately 3 weeks in macaques^51,52^. Additionally, our data suggest that the capacity to be trained extends to cells of the myeloid lineage other than monocytes: neutrophils and cDCs.

In conclusion, we assessed, for the first time, the impact of the time-interval between immunizations on the development innate and humoral responses. We shed light on the importance of the timing of immunizations in the development of a late innate myeloid response to the first immunization and hence on the quality of the innate effector response to the second immunization, which can differ from those to prime, if a sufficient amount of time passes, and its correlation with the humoral response. This is a first valuable step towards understanding the mechanisms at play during prime-boost vaccination, with the interplay of both innate and adaptive immunity, to ultimately optimize vaccines.

## Methods

### Ethics statement

The immunogenocity studies were approved by the “Ministère de l’Éducation Nationale, de l’Enseignement Supérieur et de la Recherche” (France) and the ethical committee “Comité d’éthique en expérimentation animale n°44” (France) under the reference 2015031314518254.02 (APAFIS#319). Animals were handled by veterinarians in accordance with national regulations (CEA Permit Number A 92-32-02) and the European Directive (2010/63, recommendation no. 9) and in compliance with the Standards for Human Care and Use of Laboratory of the Office for Laboratory Animal Welfare (OLAW, USA) under OLAW Assurance number #A5826-01.

### Vaccine schedule and blood sampling

Five cynomolgus macaques (males and adults imported from a breeding facility in Mauritius), identified as BC554D, BT145, CBL004, CC840, and CCB116, were immunized subcutaneously 2 weeks apart with the ANRS recombinant MVA HIV-B vaccine (MVATG17401; Transgene, Illkirch-Graffenstaden, France) at 4 × 10^8^ plaque-forming units (Fig. 1). As previously described^14–16^, this vaccine encodes the full-length *gag* sequence (amino acids 1−512), fragments of the *pol* sequence (amino acids 172−219, 325−383, and 461−519), and fragments of the *nef* sequence (amino acids 66−147 and 182−206) from the Bru/Lai isolate (Los Alamos database accession number K02013). Blood samples were longitudinally collected in lithium-heparin for soluble plasma protein quantification and single-cell mass cytometry profiling, and in ethylenediaminetetraacetic acid (EDTA) for complete blood counts. They were compared to five cynomolgus macaques, identified as BB078, BB231, BC641, BD619 and BD620, and previously immunized using the same batch of vaccine, the same route of administration, and the same dose, but according to a different schedule, with a second immunization at 2 months. Their antibody (anti-MVA IgG Ab titers using a different batch of wild-type MVA as antigen, and a different method for curve fitting and analysis, and nAb titers using a plaque reduction neutralization test (PNRT) with DF1 cells instead of a single-cycle infection assay in HeLa cells) and B-cell responses have been previously reported^14^, as well as their plasma cytokine profiles^15^, and innate myeloid cells^15^, and NK cells responses^16^.

### Serology

Wild-type MVA (MVATGN33.1 obtained from Transgene, Illkirch-Graffenstaden, France)^53^ was used to coat 96-well MaxiSorp microplates (Nunc; Thermo Fisher, Waltham USA) at 10^5^ PFU/well in coating buffer (200 mM NaHCO_3_, 80 mM Na_2_CO_3_, pH 9.5) overnight at 4 °C. Wells were washed five times with wash buffer (PBS (phosphate-buffered saline), 0.1% Tween 20, 10 mM EDTA), and blocked for 1 h at RT with 3% w/v bovine serum albumin (BSA) (Sigma). Plates were washed five times and incubated with twofold serial dilutions of macaque serum (duplicates) diluted in 1% w/v BSA in PBS for 2 h at room temperature (RT), starting at 1:50 for IgG and IgA and 1:20 for IgM. Plates were then washed five times and 1:20,000, 1:5000, or 1:1000 diluted peroxidase-conjugated goat anti-monkey H + L chain IgG (Bio-Rad, Marne-la-Coquette, France), IgA (Alpha Diagnostic, San Antonio, USA), or IgM (Bio-Rad, Hercules, USA), respectively, in 1% BSA (w/v) PBS was added and incubated for 1 h at RT. For subclasses, anti-rhesus IgG1, IgG2, or IgG3 Abs (NHP Bioresources) were biotinylated (Thermo Scientific, Waltham, USA) according to the manufacturer’s instruction. The biotinylated antibody diluted 1:500, 1:100, or 1:100, respectively, were used and incubated for 1 h at RT. Plates were washed five times and incubated with 1:1000 diluted streptavidin-biotin (Thermo Scientific, Waltham, USA) with PBS + 1% BSA for 1 h at RT. All plates were washed five times and 100 µL 3,3′,5,5′-tetramethylbenzidine (Thermo Scientific, Waltham, USA) was added and incubated for 20 min at RT in the dark. The reaction was stopped by adding 100 µL 2 N H_2_SO_4_. Absorbance was measured at 492 nm using a Spark spectrophotometer and data were analyzed using Magellan software (both from Tecan, Lyon, France). Ab titers were calculated by extrapolation from the OD as a function of a serum dilution curve (five-parameter logistic curve) and defined as the dilution of the test serum reaching 2× OD of the corresponding preimmune serum tested at a dilution of 1:50.

### Antibody neutralization assay

The presence of neutralizing antibodies was assessed using a single-cycle assay by infecting HeLa P4 cells (CD4^+^ HeLa Cells;^54^ from Pierre Charneau, Institut Pasteur, Paris, France) with MVAeGFP (MVATG15938, Transgene SA, Illkirch-Graffenstaden, France) at a multiplicity of infection of 1 after preincubating the virus with serial dilutions of heat-decomplemented serum from immunized animals (56 °C for 45 min) at 4 °C for 1 h. The percentage of eGFP^+^ cells was quantified by flow cytometry using an LSRII (BD Biosciences, Franklin Lakes, New Jersey, USA) after Blue-Vid staining (Thermofisher Scientific, Waltham, USA) and exclusion of dead cells. The sample dilution versus the percentage of eGFP^+^ (as defined using manual gating with FlowJo 10, BD Biosciences) was plotted to calculate the neutralizing antibody titer, corresponding to the inverse of the sample dilution resulting in 50% less eGFP^+^ cells observed after incubation with the paired baseline sample diluted 1:100. VIGIV (Vaccinia Immune Globulin Intravenous, BEI Resources) was used as positive control (neutralization ID50 titer of 632 ± 299).

### ELISA-based FcyRIIIa dimer-binding assay

Recombinant soluble biotin-tagged homodimers of human FcγRIIIa (CD16) were used to quantify the FcγRIIIa dimers binding capacity of MVA-specific cynomolgus macaque IgGs. Plates were coated with wild-type MVA at 10^5^ PFU/well diluted in coating buffer (200 mM NaHCO_3_, 80 mM Na_2_CO_3_, pH 9.5) overnight at 4 °C on 96-well flat-bottom MaxiSorp plates (Nunc; Thermo Fisher). The ELISA plates were then washed with PBS containing 0.05% Tween20 (Sigma-Aldrich) and blocked for 1 h at 37 °C with 200 μL PBS containing 1 mM EDTA (Sigma-Aldrich) and 1% BSA (Sigma-Aldrich). Plates were washed five times and incubated with macaque serum diluted at 1:500 in 1% w/v BSA in PBS in duplicates for 2 h at RT, starting at 1:50. Plates were then washed five times, and 0.1 mg/mL purified human FcγRIIIa-V158 ectodimer-biotin (gift from Kent SJ, Melbourne, Australia) diluted in PBS + 1% BSA + 1 mM EDTA was added to every well of the plate. Following incubation and washes, HRP-conjugated streptavidin (Thermo Scientific) was added in 1:1000 dilution with PBS + 1% BSA + 1 mM EDTA. After incubation and washes, color was developed using 3,3′,5,5′-tetramethylbenzidine (Sigma-Aldrich) followed by 1 M HCl stop solution. Absorbance at 450 nm wavelength was recorded as OD. A positive signal was defined as an OD higher than OD mean + 2× SD obtained using sera at BL.

### Quantification of cytokines, chemokines, and growth factors

Cytokines, chemokines, and growth factors present in plasma and PBMC culture supernatants were quantified as duplicates and using a multiplex immunoassay (MILLIPLEX MAP non-human primate cytokine magnetic bead panel, Millipore), and using a BioPlex 200 system as reader and the Bio-Plex data manager to analyze data (BioRad, Hercules, California, USA), except for IP-10. The IP-10 concentration was assessed by ELISA (human CXCR10/IP-10, R&D Systems).

### Whole-blood fixation

Whole-blood processing was performed as previously described^15,16,55^ to preserve all leukocytes, including granulocytes. Briefly, 1 mL blood was incubated with a fixation mixture containing PFA and glycerol^55,56^ for 10 min at 4 °C. After centrifugation, erythrocytes were lysed in 10 mL milli-Q water at RT for 20 min. Cells were then washed in 1× DPBS (Dulbecco’s phosphate-buffered saline) and stored at −80 °C at a final concentration of 15 × 10^6^ cells/mL in the fixation mixture.

### Cell staining and mass cytometry acquisitions

Cell staining was performed similarly as previously described^15^. It differed by the Ab panel and Ab batches, the addition of heparin during staining, the barcoding, and the mass cytometer used to acquire samples (CYTOF 1 versus Helios). Briefly, 3 million fixed leukocytes were thawed. After two washes with PBS + 0.5% BSA, cells were incubated with the surface antibodies at 4 °C for 30 min (Supplementary Table 3). The staining mixture contained 300 U of heparin to prevent nonspecific binding of metal by eosinophils, as suggested in the literature^20^. Samples were washed twice in 1× PBS and fixed in PBS + 1.6% PFA for 20 min RT. After permeabilization in 1× Perm/Wash Buffer (BD Biosciences) for 10 min at RT, cells were incubated with intracellular antibodies at 4 °C for 30 min. Cells were barcoded with the Cell-ID 20-Plex Pd barcoding kit (Fluidigm, South San Francisco, California, USA). In detail, after two washes in the Barcode Perm Buffer, cells were incubated with one of the indicated combinations of Pd for 30 min at RT. Finally, cells were washed in PBS and incubated overnight with 0.1 µM iridium RNA/DNA intercalator in PBS + 1.6% PFA. The next day, cells were washed three times with milli-Q water and filtered using a 35-µm nylon mesh cell strainer (BD Biosciences, Franklin Lakes, USA). EQ^TM^ four-element calibration beads (Fluidigm) were added following the manufacturer’s protocol. Sample acquisition was performed using a Helios CyTOF (Fluidigm). Five stainings/acquisitions were performed (one per animal) using the same batch of antibodies. In addition, we followed an established strategy^57^ to control the quality of each staining/acquisition and their reproducibility by including two identical control samples for each staining/acquisition (Supplementary Fig. 8).

### Mass cytometry data preprocessing and leukocyte gating

To avoid bias in density estimation by the Spanning-tree Progression Analysis of Density-normalized Events (SPADE) algorithm^58^, zero values of mean signal intensities (MSI) generated on the Helios were randomized between −1 and 0. Data were then normalized using the MATLAB normalizer from Finck et al.^59^. Samples were debarcoded using the Debarcoder software (Fluidigm, San Francisco, USA), following user guide instructions.

Initial gating was performed using Cytobank (Mountain View, California, USA) as previously described^15,55^, and included the definition of singlets (based on Ir191/Cell length), intact cells (Ir191/Ir193), no beads (Ce140/Gd155), and exclusion of CD3^+^CD66^+^ cells. Note that although the use of heparin strongly reduced the nonspecific staining of eosinophils, some CD3^+^CD66^+^ cells were still present and excluded at that step (approximately 0.2% of all acquired events).

### Automatic identification of cell populations

The SPADE algorithm was used to automatically identify cell populations as previously described^15,16^. Briefly, uniform predownsampling was used to select 95,000 cells from each sample (corresponding to the number of cells contained in the smallest sample—Supplementary Table 6). Cell clusters (groups of cells with similar phenotypic patterns) were identified using SPADE, and applied to the whole set of samples (all macaques and all timepoints). Upsampling was eventually performed.

The optimal SPADE settings for this particular dataset were determined using the SPADEVizR package^60^. The optimal SPADE analysis was obtained using 28 clustering markers (CADM1, CCR5, CCR7, CD1c, CD3, CD4, CD8, CD11a, CD11b, CD11c, CD14, CD16, CD20, CD23, CD32, CD39, CD45, CD62L, CD64, CD66, CD86, CD123, CD125, CD141, CD172a, CXCR4, FcεRI, and HLA-DR), a density-based downsampling of 10%, an outlier density parameter of 0.01, and by identifying 800 clusters. The quality of the SPADE clustering was quantified as the percentage of clusters displaying a unimodal and narrow distribution for all clustering markers. Marker distributions were assessed using the Hartigan’s dip test (*p* value < 0.05 to reject the unimodality hypothesis). Marker distributions with an interquartile range (IQR) < 2 were considered to be narrow. These settings resulted in a clustering quality of 80.12% uniform clusters. The numbers and percentages of nonuniform clusters are shown for each marker in Supplementary Table 7.

### Leukocyte counts, absolute number calculation, and abundance profiles

Leukocyte counts were quantified using an HMX instrument (Beckman Coulter, Brea, California, USA). The absolute number of cells in a population was computed as *N* = the absolute number of leukocytes expressed per μL of blood × number of cells in the population detected by the CyTOF/the total number of leukocytes (defined as non-CD3^+^CD66^+^ cells) detected by the CyTOF. The kinetics of the cell absolute number was designated as the abundance profile. The complete blood count was not available for samples at D28PB.

### Heatmap representations of the cell cluster phenotypes

Categorical heatmaps showing the cell cluster phenotypes were generated using SPADEVizR^60^. The expression range of each marker was divided between the 5th and the 95th percentiles into five categories for all cell clusters. The mean of the median MSI for each marker among samples was mapped onto these five categories to infer the cell cluster phenotype and annotate it. For each cluster, samples contributing less than ten cells were excluded for cell cluster phenotype inference. Hierarchical clusterings of cell clusters and markers, represented in the heatmaps, were performed using the Euclidean metric based on the ward.D linkage method.

### Phenotypic and kinetic families

Cell clusters sharing similar phenotypes were grouped into phenotypic families based on the cluster dendrogram resulting from the hierarchical clustering computed on phenotypic categories of marker expression. Phenotypic families sharing similar dynamics were grouped into kinetic families based on their abundance profiles. This determination was performed using the hierarchical method based on the Pearson correlation coefficient and complete linkage method.

### Statistical tests

Blood protein concentrations and cell abundances were compared between timepoints using the two-sided permutation test available in the “exactRankTests” R package (available at https://cran.r-project.org/web/packages/exactRankTests/index.html). The AUC corresponds to the sum over time of all plasma soluble factor concentrations (cumulated concentration) or cell abundances (cumulated abundance) between H6PP and D14PP and H6PB and D14PB. AUC were compared using a permutation test.

### Discrimination between innate myeloid responses to first and second immunization

The Least Absolute Shrinkage and Selection Operator (LASSO) approach was performed in R using the “lars” package. Centered and reduced abundance profiles of kinetic families were used as entry parameters. The validity of classification at each iteration was assessed by cross-validation.

### Phenotypic comparison of cell clusters from the two vaccine schedules

Cell cluster phenotypes from this study (shortened vaccine schedule) and our previous study (longer vaccine schedule)^15^ were compared using the Manhattan distance and visualized using the CytoCompare package^61^. Distances were computed based on the heatmap expression categories of the 27 markers shared between both panels (CCR5, CCR7, CD3, CD4, CD8, CD11a, CD11b, CD11c, CD14, CD16, CD20, CD23, CD32, CD45, CD64, CD66, CD86, CD123, CXCR4, HLA-DR, IFNα, IL-1α, IL-4, IL-6, IL-8, IL-12, and IP-10). Distances consisted of the sum of the absolute value of the difference between the categorical value of each cluster for each marker. Two clusters were considered to be associated if this sum, calculated for the 27 markers, was ≤9 and no term was >2 (if one term was >2, the distance was penalized to be >10).

### Correlations between antibody response and innate myeloid responses

Only associated cell populations between the two studies and defined as the closest neighbor of each other based on the ratio between the actual number of associations (clusters linked together) and the number of potential associations between them were used in the correlation analysis. The AUC was used to assess the magnitude of the response to prime (Baseline, H6, D1, D3, and D8 post-prime (PP)) and early or delayed boost (H0, H6, D1, D3, and D8 post-boost (PB)). To avoid technical bias in this calculation, the area was normalized based on the mean abundance of each cell population within each schedule during the response to prime. In other words, for a given animal and a given cell population: AUC = sum of the abundances during prime (or boost)/average abundance during the prime for all animals from the same vaccine schedule. Pearson’s correlation was computed between the AUC of each innate myeloid cell population abundance and the antibody response at its peak (either D8PB or D14PB, according to the response) and in the long term (at M6PB).

### PBMC culture

PBMCs were isolated after Ficoll (Lymphocyte Separation Media 1077, GE Healthcare, Chicago, USA) diluted to 95% in 1× PBS and cultured at 3× 10^6^/200µL in RPMI-1640 (Invitrogen, Carlsbad, USA), containing 10% heat-inactivated fetal calf serum (Eurobio, Courtaboeuf, France) and 1% penicillin-streptomycin/neomycin (Thermo Fisher Scientific,Waltham, USA) in 96-well flat-bottom plate for 18 h at 37 °C without re-stimulation. Supernatants were harvested after centrifugation at 300 × *g* for 2 min and assessed for the presence of inflammatory cytokines.

### Reporting summary

Further information on research design is available in the Nature Research Reporting Summary linked to this article.

## Supplementary information

Reporting Summary

Supplementary Information

## Data Availability

Mass cytometry data are available on the FlowRepository database through ID FR-FCM-Z28T. An interactive interface can be found on the IDMIT data dissemination platform (available at http://data.idmitcenter.fr/MVA-innate-myeloid-early-boost/)
