# Supplementary material for: Innate and secondary humoral responses are improved by increasing the time between MVA vaccine immunizations
Source: NPJ Vaccines. 2020 Mar 19;5:24. doi: 10.1038/s41541-020-0175-8 (PMC7081268; doi:10.1038/s41541-020-0175-8)
Supplement: Supplementary file 2 — Supplementary Information [file 41541_2020_175_MOESM2_ESM.pdf]

Innate and secondary humoral responses are improved by increasing the time between MVA vaccine immunizations

Jean-Louis Palgen[1,2], Nicolas Tchitchek[1,2], André Rodriguez-Pozo[1,2], Quentin Jouhault[1,2], Hadjer Abdelhouahab[1,2], Nathalie Dereuddre-Bosquet[1,2], Vanessa Cont"reras[1,2], Frédéric Martinon[1,2], Antonio Cosma[1,2], Yves Lévy[2,3], Roger Le Grand[1,2], and Anne-Sophie Beignon[1,2*]

[1] CEA – Université Paris Sud 11 – INSERM U1184, Immunology of Viral Infections and Autoimmune Diseases, IDMIT department, IBFJ, 92265 Fontenay-aux-Roses, France

[2] Vaccine Research Institute, Henri Mondor Hospital, 94010 Créteil, France

[3] Institut Mondor de Recherche Biomédicale – INSERM U955, Eq.16, 94010, Créteil, France

*Corresponding author and lead contact: Anne-Sophie Beignon; 18, route du Panorama, 92265 Fontenay-aux-Roses, France; Phone: +33 1 46 54 80 27, Fax: +33 1 46 54 77 26; email: anne-sophie.beignon@cea.fr

Supplementary material

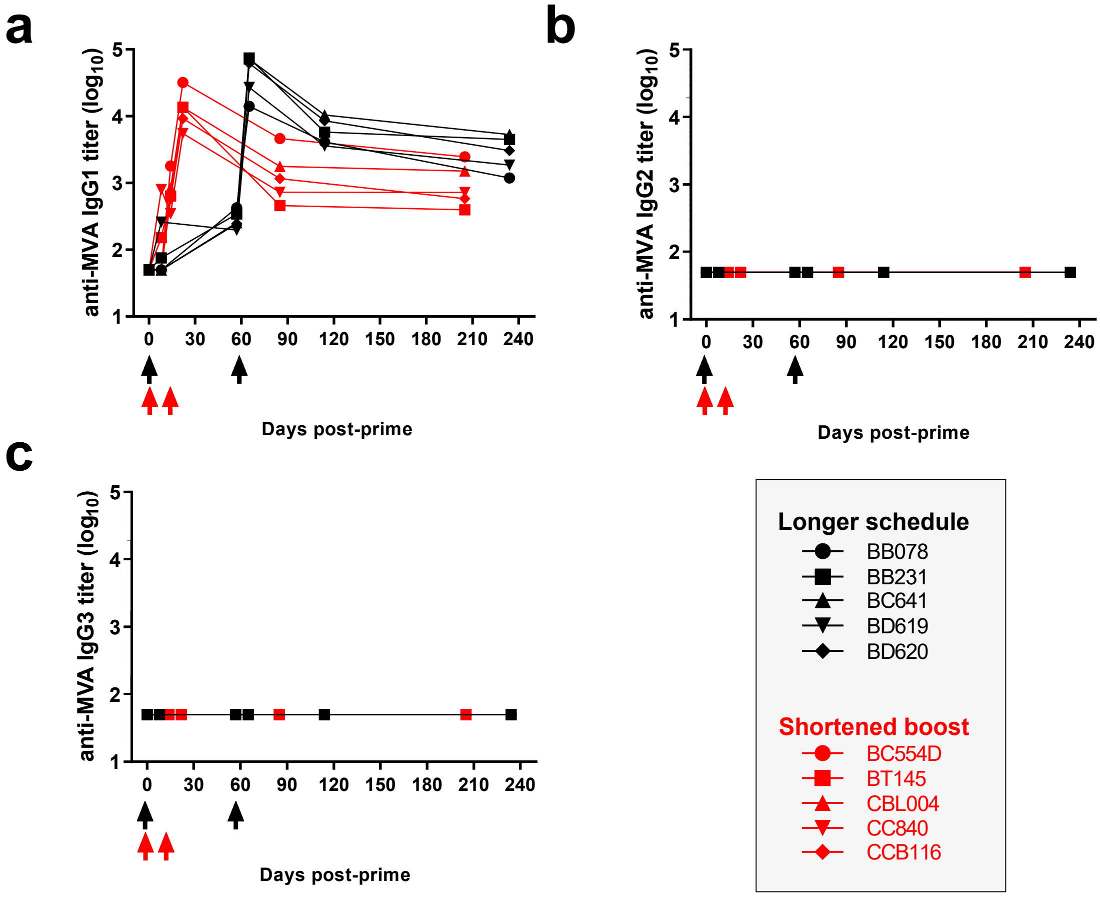

Supplementary Figure 1 – MVA-specific IgG subclasses in serum over time. MVA-specific (**a**) IgG1, (**b**) IgG2, and (**c**) IgG3 titers were measured by direct ELISA during the prime/early boost (red) or prime/classical boost (black) vaccination schedule. Individual titers are shown. Immunizations are indicated by arrows.

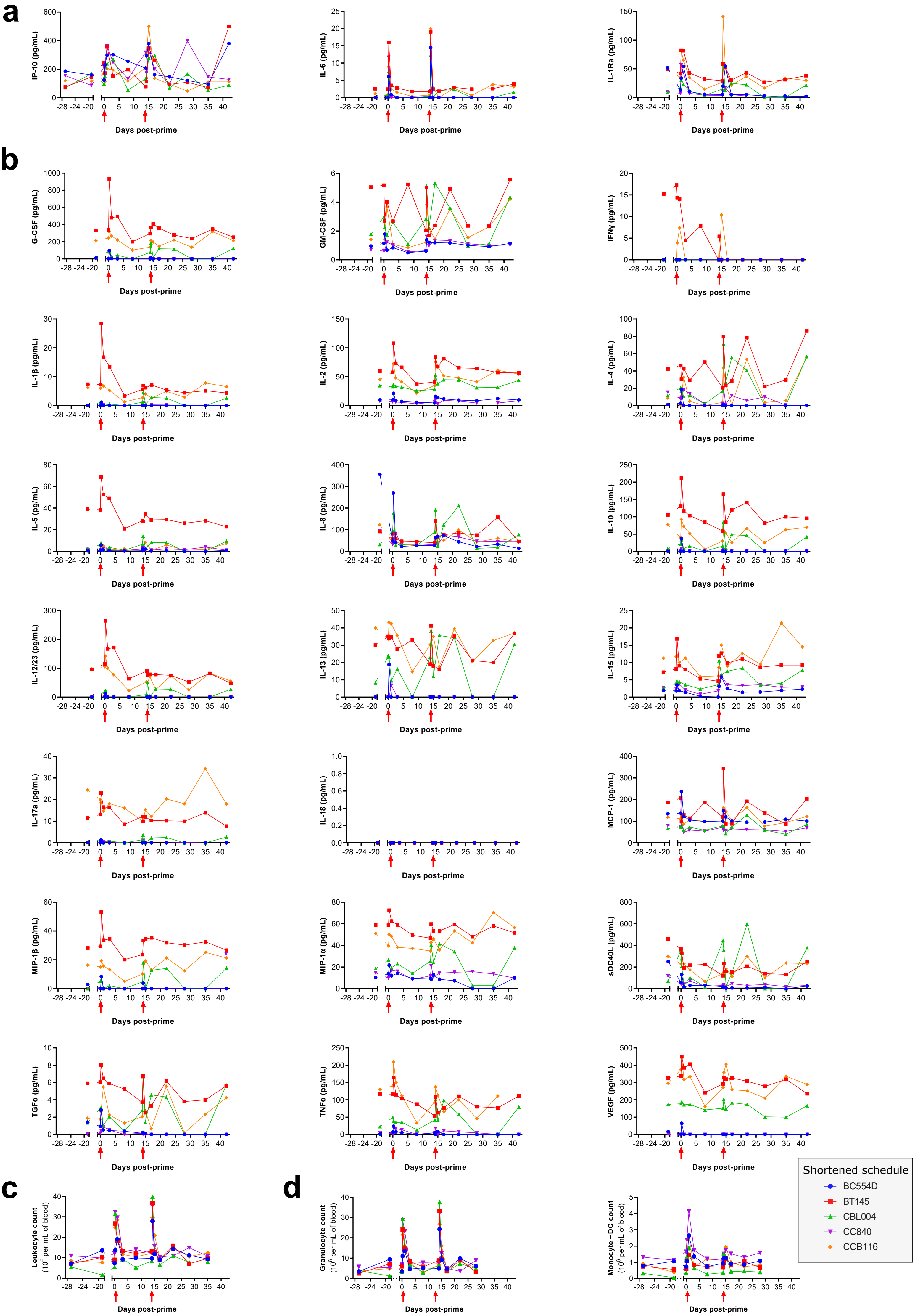
a
b
c
d
IP-10 (pg/mL)
IL-6 (pg/mL)
IL-1Ra (pg/mL)
G-CSF (pg/mL)
GM-CSF (pg/mL)
IFNγ (pg/mL)
IL-1β (pg/mL)
IL-2 (pg/mL)
IL-4 (pg/mL)
IL-5 (pg/mL)
IL-8 (pg/mL)
IL-10 (pg/mL)
IL-12/23 (pg/mL)
IL-13 (pg/mL)
IL-15 (pg/mL)
IL-17a (pg/mL)
IL-18 (pg/mL)
MCP-1 (pg/mL)
MIP-1β (pg/mL)
MIP-1α (pg/mL)
sDC40L (pg/mL)
TGFα (pg/mL)
TNFα (pg/mL)
VEGF (pg/mL)
Leukocyte count (10^6 per mL of blood)
Granulocyte count (10^6 per mL of blood)
Monocyte - DC count (10^6 per mL of blood)
Days post-prime
Shortened schedule
BC554D
BT145
CBL004
CC840
CCB116

Supplementary Figure 2 – Plasma cytokine concentrations and blood leukocyte counts following prime/early boost. (**a**) Cytokines significantly impacted by vaccination. (**b**) Cytokines not significantly impacted by vaccination. (**c**) Leukocyte counts after vaccination. (**d**) Granulocyte and Monocyte-DC counts after vaccination. (**a-d**) The individual curves are displayed. Immunizations are indicated by red arrows.

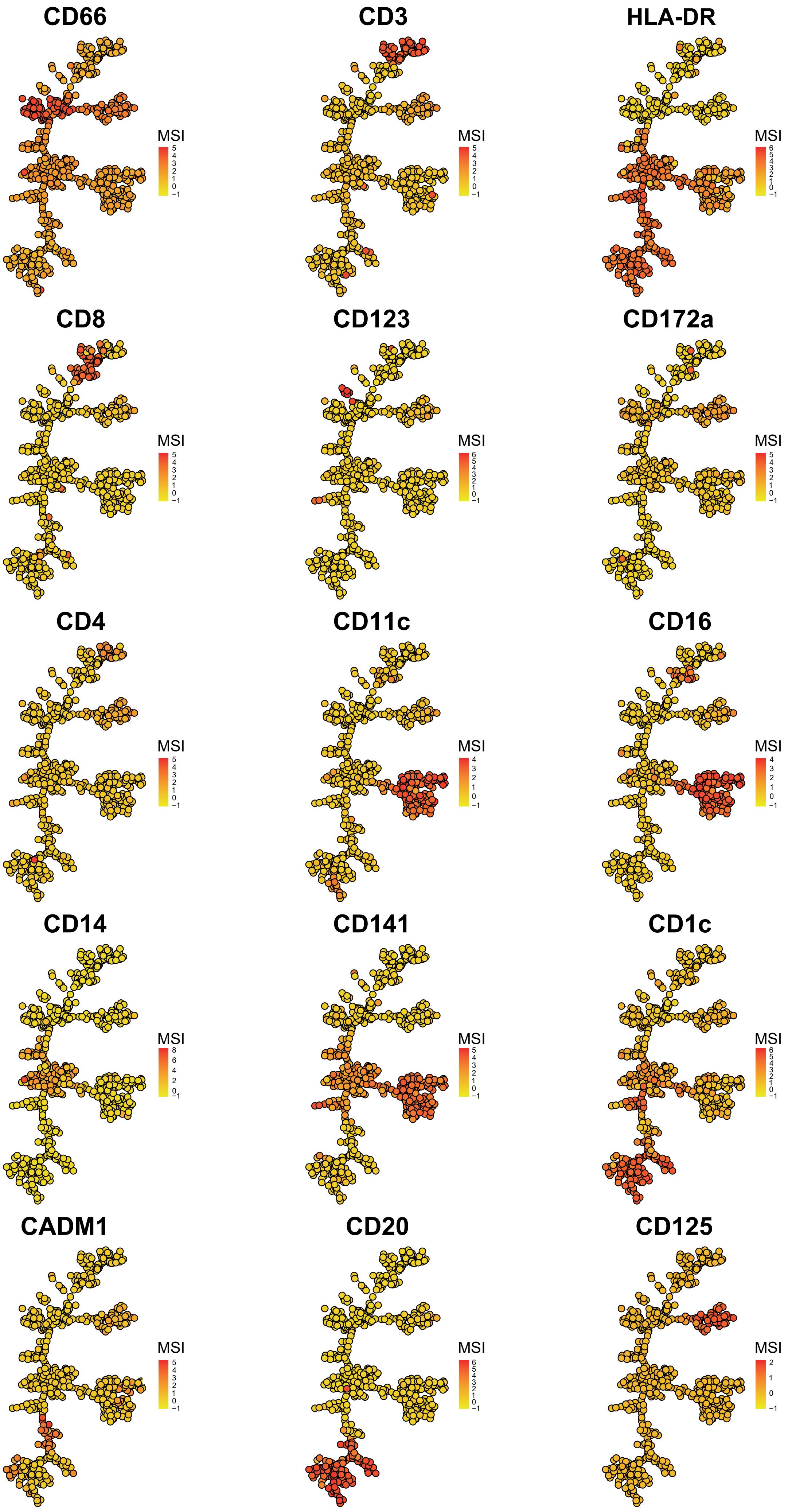
CD66
CD3
HLA-DR
CD8
CD123
CD172a
CD4
CD11c
CD16
CD14
CD141
CD1c
CADM1
CD20
CD125
MSI

Supplementary Figure 3 – SPADE tree annotation. The topology of the SPADE tree is displayed, with each node colored by the expression of the 15 markers used to annotate cell clusters.

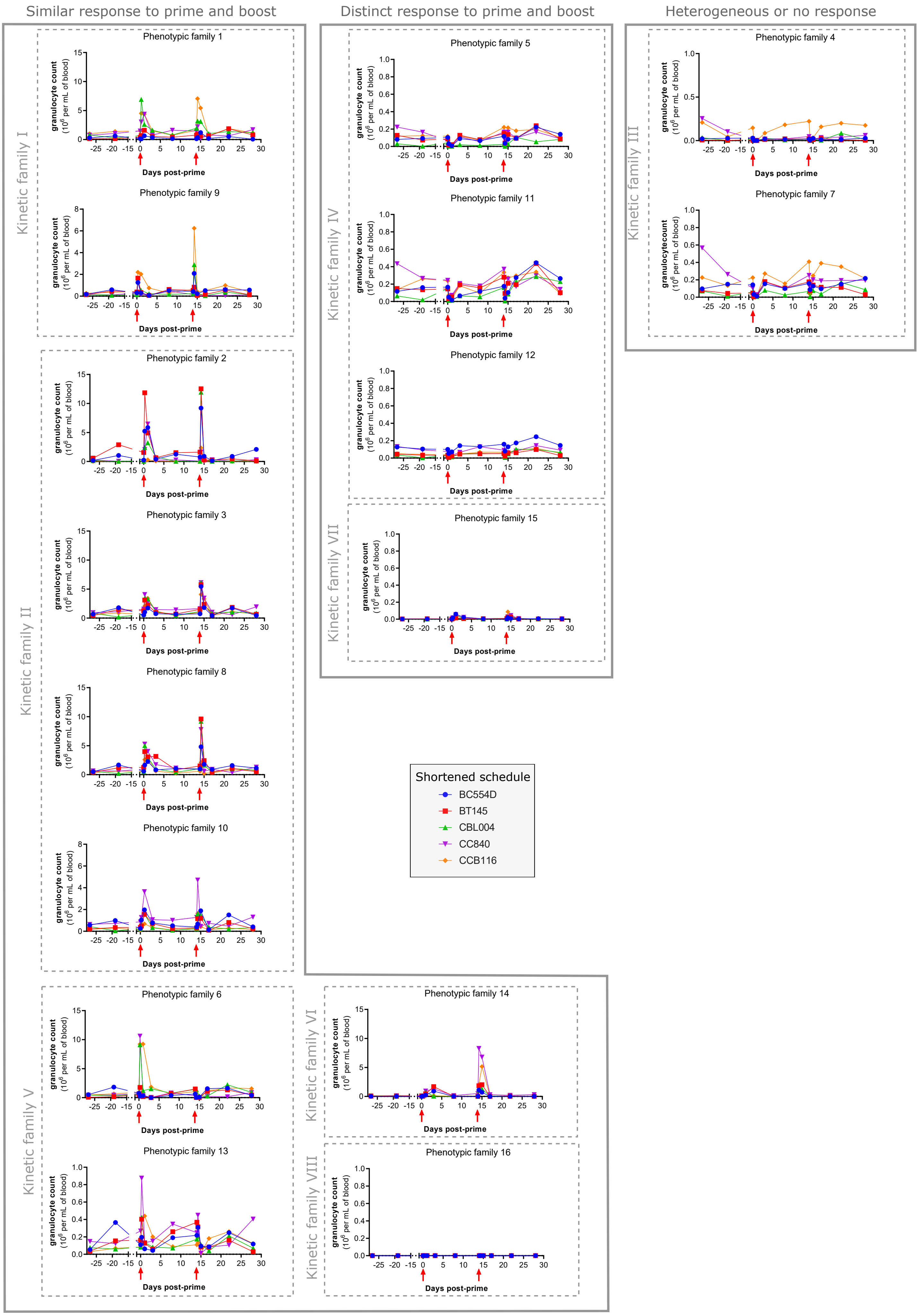

Similar response to prime and boost
Distinct response to prime and boost
Heterogeneous or no response
Kinetic family I
Kinetic family II
Kinetic family III
Kinetic family IV
Kinetic family V
Kinetic family VI
Kinetic family VII
Kinetic family VIII
Phenotypic family 1
Phenotypic family 2
Phenotypic family 3
Phenotypic family 4
Phenotypic family 5
Phenotypic family 6
Phenotypic family 7
Phenotypic family 8
Phenotypic family 9
Phenotypic family 10
Phenotypic family 11
Phenotypic family 12
Phenotypic family 13
Phenotypic family 14
Phenotypic family 15
Phenotypic family 16
granulocyte count (10^6 per mL of blood)
Days post-prime
Shortened schedule
BC554D
BT145
CBL004
CC840
CCB116

Supplementary Figure 4 – Abundance of granulocyte phenotypic families. The absolute count of each of the 16 granulocytes phenotypic families are displayed. Dotted grey frames regroup phenotypic families into their associated kinetic families and bold grey frames regroup kinetic families into superkinetic families. Kinetic families are ordered with respect to **Figure 5**.

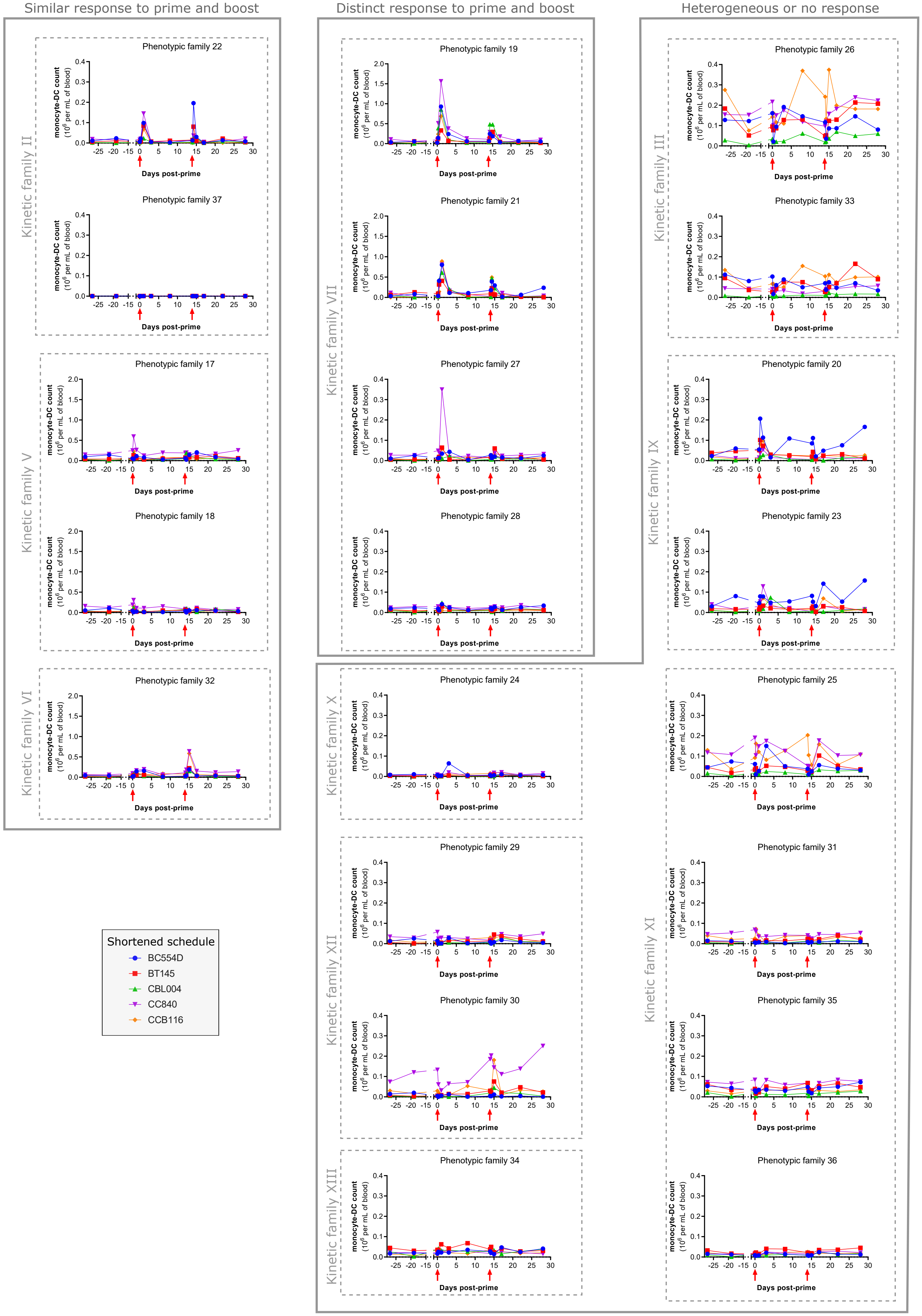
Similar response to prime and boost
Distinct response to prime and boost
Heterogeneous or no response
Kinetic family II
Kinetic family V
Kinetic family VI
Kinetic family VII
Kinetic family X
Kinetic family XII
Kinetic family XIII
Kinetic family III
Kinetic family IX
Kinetic family XI
Phenotypic family 22
Phenotypic family 37
Phenotypic family 17
Phenotypic family 18
Phenotypic family 32
Phenotypic family 19
Phenotypic family 21
Phenotypic family 27
Phenotypic family 28
Phenotypic family 24
Phenotypic family 29
Phenotypic family 30
Phenotypic family 34
Phenotypic family 26
Phenotypic family 33
Phenotypic family 20
Phenotypic family 23
Phenotypic family 25
Phenotypic family 31
Phenotypic family 35
Phenotypic family 36
monocyte-DC count
(10^6 per mL of blood)
Days post-prime
Shortened schedule
BC554D
BT145
CBL004
CC840
CCB116

Supplementary Figure 5 – Abundance of monocytes-DCs phenotypic families. The absolute counts of each of the 21 monocytes-DCs phenotypic families are displayed. Dotted grey frames regroup phenotypic families into their associated kinetic families and bold grey frames regroup kinetic families into superkinetic families. Kinetic families are ordered with respect to **Figure 5**.

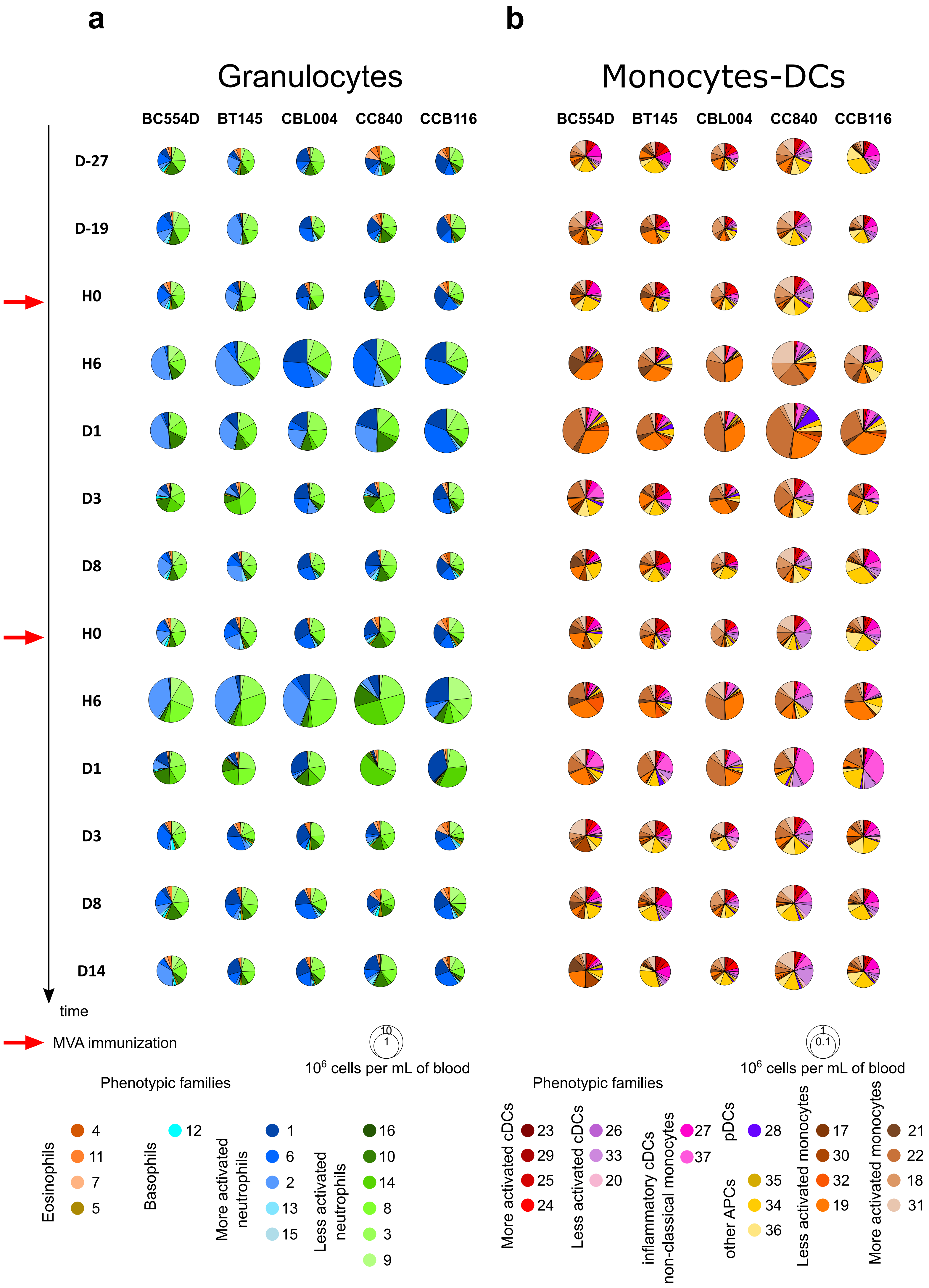

a
b
Granulocytes
Monocytes-DCs
BC554D
BT145
CBL004
CC840
CCB116
D-27
D-19
H0
H6
D1
D3
D8
H0
H6
D1
D3
D8
D14
time
MVA immunization
10
1
10^6 cells per mL of blood
1
0.1
10^6 cells per mL of blood
Phenotypic families
Eosinophils
4
11
7
5
Basophils
12
More activated neutrophils
1
6
2
13
15
Less activated neutrophils
16
10
14
8
3
9
Phenotypic families
More activated cDCs
23
29
25
24
Less activated cDCs
26
33
20
inflammatory cDCs non-classical monocytes
27
37
pDCs
28
other APCs
35
34
36
Less activated monocytes
17
30
32
19
More activated monocytes
21
22
18
31

Supplementary Figure 6 – Phenotypic composition after prime/early boost. The composition in (**a**) granulocytes, and (**b**) monocytes-DCs phenotypic families along the timecourse of vaccination. Cell abundance is given by the size of the pie chart. The color-code and annotation for each phenotypic family is conserved between pie-slices, heatmaps (**Figure 4**), and circos (**Figure 5**).

a

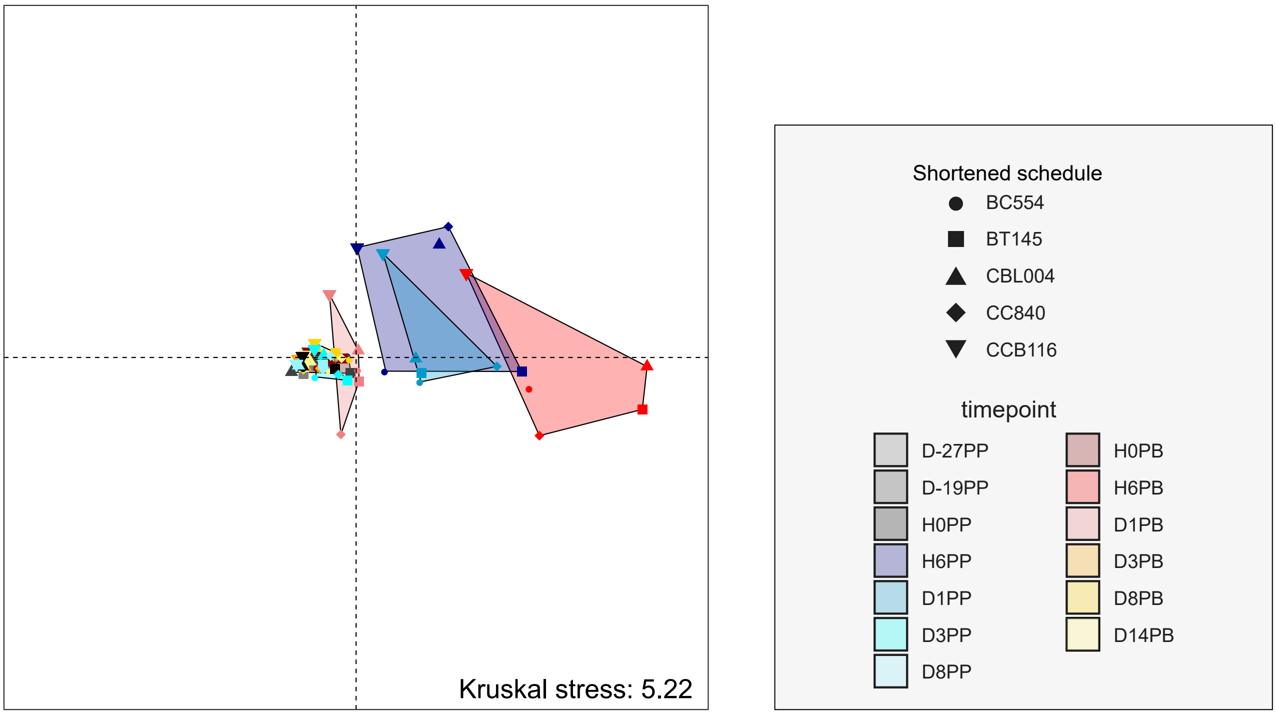

b

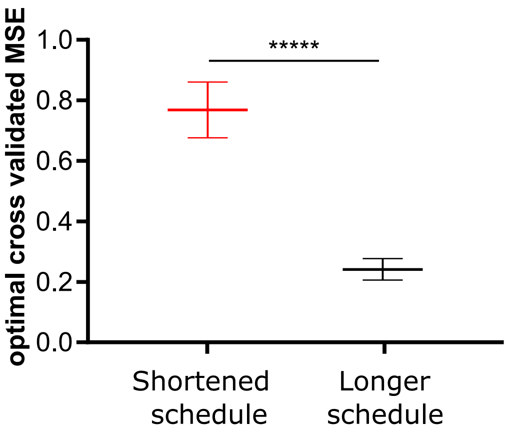

Supplementary Figure 7 – Comparison of the innate myeloid cell response to prime and to early boost. (**a**) Multidimensional Scaling (MDS) representation was calculated based on the cell abundances of the kinetic families. The proportion of information lost during the dimensionality reduction process is indicated with the Kruskal Stress. Samples collecting at the same timepoint were delineated using convex hulls, and color-coded. (**b**) The Least Absolute Shrinkage and Selection Operator (LASSO) approach was applied to discriminate samples from post-prime and post-early boost responses, and the mean square error (MSE) of the optimal LDA obtained on the prime/early boost dataset was compared that of the prime/classical boost dataset. *****, p-value <0.00001 using a t-test.

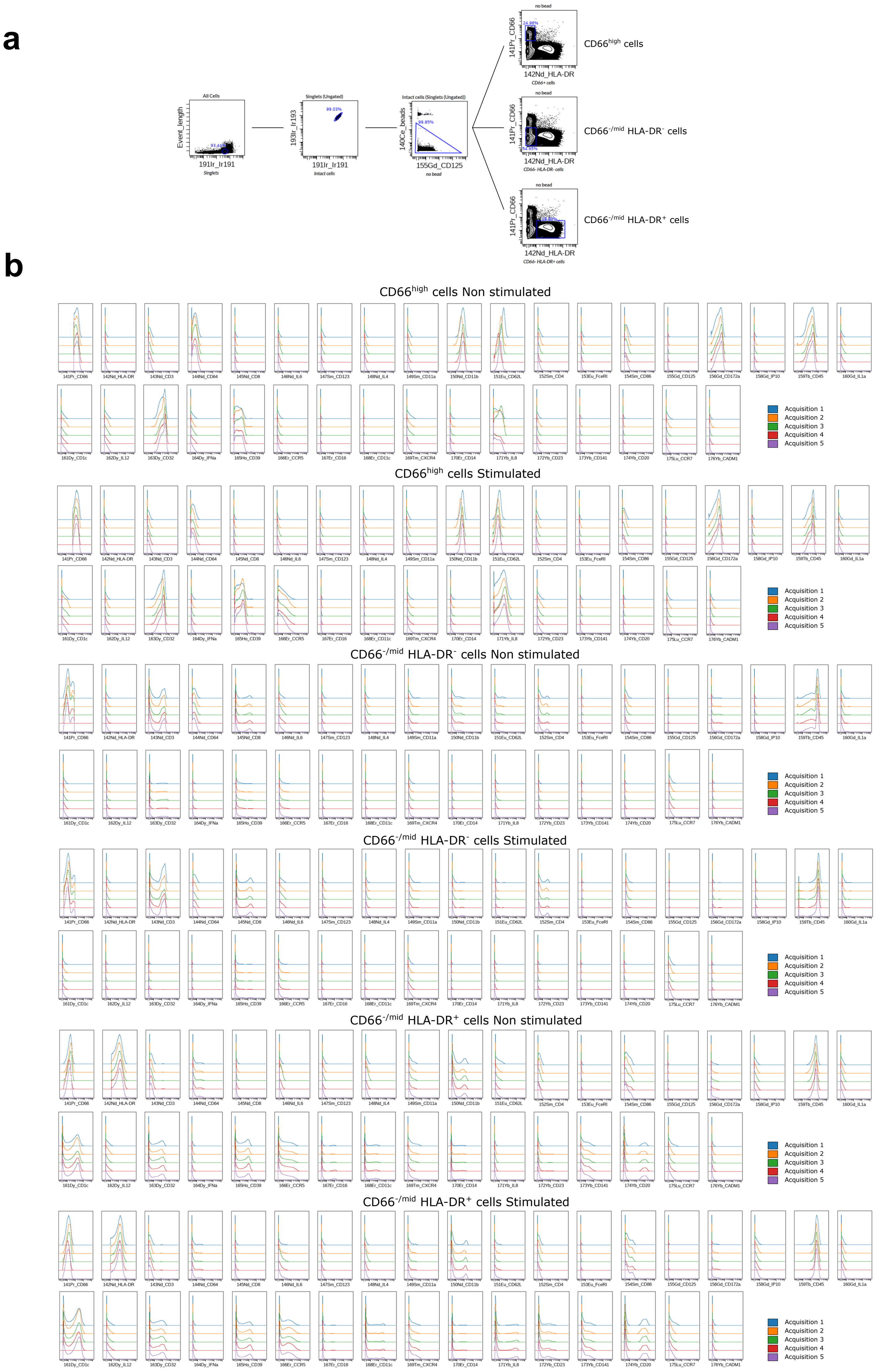
a
All Cells
Event_length
191Ir_Ir191
Singlets
Singlets (Ungated)
193Ir_Ir193
Intact cells
Intact cells (Singlets (Ungated))
140Ce_beads
155Gd_CD125
no bead
141Pr_CD66
142Nd_HLA-DR
CD66high cells
CD66-/mid HLA-DR- cells
CD66-/mid HLA-DR+ cells
b
CD66high cells Non stimulated
CD66high cells Stimulated
CD66-/mid HLA-DR- cells Non stimulated
CD66-/mid HLA-DR- cells Stimulated
CD66-/mid HLA-DR+ cells Non stimulated
CD66-/mid HLA-DR+ cells Stimulated
Acquisition 1
Acquisition 2
Acquisition 3
Acquisition 4
Acquisition 5

Supplementary Figure 8 – Staining and acquisition controls. The same control samples were stained and acquired for each of the five stainings-acquisitions of the samples from the vaccinated animals. These controls were obtained after *ex vivo* restimulation of whole blood from one unrelated macaque with or without a TLR ligand cocktail (TLR 3, 4,7,and 8) composed of 1 µg/mL LPS (from *E. coli0111:B4*, Invivogen), 100 µg/mL Poly I-C (Invivogen),and 10 µg/mL R848 (Mabtech). Whole blood was incubated with or without stimulation for 6h, with the addition of 10 µg/mL BFA (Sigma) during the last 4h. Cells were then fixed, red-blood cells lysed, and frozen as for vaccinated animal samples. The staining and acquisition of the control samples were performed in parallel of those of the vaccinated animals samples in the following order: acquisition 1 corresponding to BC554D, 2 to BT145, 3 to CBL004, 4 to CC840 and 5 to CCB116. (**a**) Gating strategy to define the cell populations to compare staining profiles between control samples. The non stimulated control sample for the acquisition of macaque BC554D samples is shown. (**b**) Distribution of all markers across $CD66^{high}$, $CD66^{-/mid}$ $HLA\text{-}DR^{-}$,and $CD66^{-/mid}$ $HLA\text{-}DR^{+}$ cells.

		IgG	IgA	nAb	FcγRIIIa dimer binding
prime/early boost	baseline vs D8PP	1,0000	0,4444	1,0000	\
	baseline vs D14PP	0,0079	0,1667	1,0000	0,2063
	baseline vs D8PB	0,0079	0,0079	0,0476	\
	baseline vs D14PB	0,0079	0,1667	0,0476	0,0079
	baseline vs D28PB	0,0079	0,0079	0,0476	\
	baseline vs D57PB	0,0079	0,0079	0,0476	0,0238
	baseline vs M3PB	0,0079	0,0476	0,1667	\
	baseline vs M6PB	0,0079	0,0476	0,1667	0,0238
	D8PP vs D14PP	0,0079	0,3651	1,0000	\
	D8PP vs D8PB	0,0079	0,0238	0,0476	\
	D8PP vs D14PB	0,0079	0,2063	0,0476	\
	D8PP vs D28PB	0,0079	0,0714	0,0476	\
	D8PP vs D57PB	0,0079	0,2937	0,0476	\
	D8PP vs M3PB	0,0079	0,1032	0,1667	\
	D8PP vs M6PB	0,0079	0,1270	0,1667	\
	D14PP vs D8PB	0,0079	0,1111	0,0476	\
	D14PP vs D14PB	0,0079	0,4048	0,0476	0,0079
	D14PP vs D28PB	0,0159	0,5317	0,0476	\
	D14PP vs D57PB	0,1349	0,9762	0,0476	0,1111
	D14PP vs M3PB	0,4603	0,9444	0,1667	\
	D14PP vs M6PB	0,5714	1,0000	0,1667	\
	D8PB vs D14PB	0,4921	0,5238	0,7063	\
	D8PB vs D28PB	0,0476	0,2619	1,0000	\
	D8PB vs D57PB	0,0079	0,0714	0,3651	\
	D8PB vs M3PB	0,0079	0,1667	0,1270	\
	D8PB vs M6PB	0,0079	0,1349	0,1032	0,1667
	D14PB vs D28PB	0,0794	0,7937	0,8571	\
	D14PBvs D57PB	0,0079	0,2937	0,4683	0,0079
	D14PB vs M3PB	0,0079	0,4444	0,1270	\
	D14PB vs M6PB	0,0079	0,4524	0,1032	0,0079
	D28PB vs D57PB	0,0079	0,3810	0,5556	\
	D28PB vs M3PB	0,0079	0,4286	0,3810	\
	D28PB vs M6PB	0,0079	0,3333	0,3333	\
	D57PB vs M3PB	0,1349	0,7460	0,5397	\
	D57PB vs M6PB	0,1032	1,0000	0,5714	0,5556
	M3PB vs M6PB	0,6190	0,8016	0,8571	\
rime/delayed boos	baseline vs D8PP	0,4444	0,0476	1,0000	\
	baseline vs D14PP	0,0079	0,0476	1,0000	0,0952
	baseline vs D8PB	0,0079	0,0476	0,0079	\
	baseline vs D14PB	0,0079	0,0476	0,0079	0,0079
	baseline vs D28PB	0,0476	0,0079	0,0079	\
	baseline vs D57PB	0,0079	0,0079	0,0079	0,0079
	baseline vs M3PB	0,0079	0,0079	0,0079	\
	baseline vs M6PB	0,0079	0,0079	0,0079	0,0397
	D8PP vs D14PP	0,0079	0,0556	1,0000	\
	D8PP vs D8PB	0,0079	0,0476	0,0079	\
	D8PP vs D14PB	0,0079	0,0476	0,0079	\
	D8PP vs D28PB	0,0476	0,0079	0,0079	\
	D8PP vs D57PB	0,0079	0,0079	0,0079	\
	D8PP vs M3PB	0,0079	0,0079	0,0079	\
	D8PP vs M6PB	0,0079	0,0079	0,0079	\
	D14PP vs D8PB	0,0079	0,0476	0,0079	\
	D14PP vs D14PB	0,0079	0,0476	0,0079	0,0079
	D14PP vs D28PB	0,1429	0,0079	0,0079	\
	D14PP vs D57PB	0,0159	0,0714	0,0079	0,0079
	D14PP vs M3PB	0,0159	0,1032	0,0079	\
	D14PP vs M6PB	0,0397	0,0079	0,0079	\
	D8PB vs D14PB	0,7460	1,0000	0,7619	\
	D8PB vs D28PB	0,0238	0,4048	0,2222	\
	D8PB vs D57PB	0,0079	0,1111	0,0079	\
	D8PB vs M3PB	0,0079	0,1032	0,0079	\
	D8PB vs M6PB	0,0079	0,4921	0,0079	0,0159
	D14PB vs D28PB	0,0238	0,4127	0,2302	\
	D14PBvs D57PB	0,0079	0,0873	0,0079	0,0079
	D14PB vs M3PB	0,0079	0,0873	0,0079	\
	D14PB vs M6PB	0,0079	0,5873	0,0079	0,0079
	D28PB vs D57PB	0,9206	0,1587	0,0079	\
	D28PB vs M3PB	0,6587	0,2222	0,0079	\
	D28PB vs M6PB	0,3968	0,1190	0,0079	\
	D57PB vs M3PB	0,5079	0,6349	0,6032	\
	D57PB vs M6PB	0,2460	0,0317	0,9841	0,0159
	M3PB vs M6PB	0,4921	0,0238	0,6587	\

Supplementary Table 1 – Statistics for antibody titers. P-values of the permutation tests are given for the indicated comparisons. Red indicate significant differences, $p < 0.05$.

Cytokine	H6PP vs H0PP	D1PP vs H0PP	D3PP vs H0PP	D8PP vs H0PP	H0PB vs H0PP	H6PB vs H0PP	D1PB vs H0PP	D3PB vs H0PP	D8PB vs H0PP	D14PB vs H0PP	D28PB vs H0PP	H6PP vs H6PB	D1PP vs D1PB	D3PP vs D3PB	D8PP vs D8PB	D14PP vs D14PB
IP-10	0,8810	0,0476	0,1508	0,7302	0,9365	0,4286	0,0079	0,4603	0,0556	1,0000	0,0714	0,9365	0,6190	0,1508	0,7381	0,2937
G-CSF	0,5952	0,8889	1,0000	0,4841	0,8413	0,9206	1,0000	1,0000	1,0000	0,6032	0,9524	0,8413	0,6349	0,7619	0,8413	0,4444
GM-CSF	0,8730	1,0000	0,8016	0,7937	0,5635	0,3095	0,7222	1,0000	0,5476	0,6429	0,4127	0,5635	0,1667	0,4524	0,6905	0,3730
IFNγ	1,0000	1,0000	1,0000	1,0000	1,0000	1,0000	1,0000	1,0000	1,0000	1,0000	1,0000	1,0000	0,7222	0,7222	1,0000	1,0000
IL10	0,5159	1,0000	0,9206	0,5635	0,6429	0,5714	0,8095	1,0000	0,7778	0,6429	1,0000	0,6429	0,8333	0,8413	0,9048	0,4048
IL-12/23	0,5952	1,0000	1,0000	0,3651	0,6349	0,7143	0,6032	0,5238	0,6825	0,3651	0,4921	0,6349	0,5159	0,5635	0,7222	0,3651
IL-13	0,7619	1,0000	0,8571	0,3651	0,6667	0,7143	0,6190	0,7143	0,8095	0,2857	0,7619	0,6667	1,0000	0,6667	1,0000	0,2857
IL-15	0,4762	1,0000	0,9206	0,3095	0,3889	0,4286	0,2857	0,7540	0,5556	0,9048	0,6032	0,3889	0,9206	0,2540	0,7381	0,1349
IL-17a	0,7619	0,8413	1,0000	0,7619	0,6825	0,7619	0,7619	0,6825	1,0000	0,7619	0,8413	0,6825	0,4365	0,8413	0,6825	0,7619
IL-18	1,0000	1,0000	1,0000	1,0000	1,0000	1,0000	1,0000	1,0000	1,0000	1,0000	1,0000	1,0000	1,0000	1,0000	1,0000	1,0000
IL-1β	0,7222	0,8810	0,9206	0,3651	0,8095	0,8730	0,8413	1,0000	1,0000	0,5238	1,0000	0,8095	0,7222	0,7222	0,8016	0,2857
IL-1Ra	0,0635	0,0476	0,8016	0,2063	0,5476	0,2381	0,2460	0,5476	0,9444	0,1905	0,7381	0,5476	0,8651	0,4286	0,6746	0,3254
IL-2	0,5476	1,0000	0,9524	0,2857	0,5635	0,5635	0,8413	0,7381	1,0000	0,9365	1,0000	0,5635	1,0000	0,8333	0,6825	0,2460
IL-4	0,8492	0,7698	0,7937	0,6032	0,5714	0,1984	1,0000	0,7937	0,3095	0,5397	0,2778	0,5714	0,2222	0,5397	0,6667	0,2063
IL-5	0,7222	0,8810	0,8810	0,7222	0,8810	0,7619	1,0000	1,0000	1,0000	0,7222	1,0000	0,8810	0,8016	0,8810	0,8810	0,5159
IL-6	0,0079	0,3810	0,9206	0,5635	0,7143	0,0159	0,9206	1,0000	0,4286	0,8413	0,3968	0,7143	0,3968	0,6190	0,9206	0,1667
IL-8	0,2698	0,9206	0,1111	0,0794	0,4683	0,3571	0,7143	0,1984	0,1984	0,4841	0,7540	0,4683	0,7540	0,7143	0,0079	0,0159
MCP-1	0,7222	0,3095	0,3889	0,6429	0,4206	0,6270	0,4365	0,3254	0,8730	0,2857	1,0000	0,4206	0,5397	1,0000	0,6825	0,4048
MIP-1α	0,8571	1,0000	1,0000	0,6905	0,7778	0,7937	1,0000	1,0000	0,8810	0,5317	0,8333	0,7778	1,0000	0,9286	1,0000	0,4921
MIP-1β	0,6190	1,0000	1,0000	0,6429	0,8254	0,7540	1,0000	0,7619	0,7937	0,8413	0,8730	0,8254	0,7937	1,0000	1,0000	0,3254
sCD40L	0,8730	0,2222	0,2540	0,0952	0,5635	0,6190	0,1508	0,2302	1,0000	0,1111	0,7381	0,5635	0,6825	0,6190	0,6508	0,3254
TGFα	0,6429	0,8730	1,0000	0,5794	0,6667	0,5714	0,4762	0,6667	0,7143	0,2460	0,7381	0,6667	1,0000	0,3413	0,7302	0,5000
TNFα	0,6429	1,0000	0,8333	0,2857	0,6667	0,9206	0,7381	0,7857	0,9444	0,3095	1,0000	0,6667	0,8810	0,7302	1,0000	0,3492
VEGF	0,7381	0,9048	1,0000	0,4921	0,7619	1,0000	1,0000	0,9048	0,8095	0,6032	0,6825	0,7619	0,7302	1,0000	0,9048	0,6667

Supplementary Table 2 – Statistics for cytokines concentrations. P-values of the permutation tests are given for the indicated comparisons. Red indicate significant differences, p <0.05.

Metal	Marker	Clone	Supplier	Catalog number	Lot number	Internal batch number	Surface	Intra-cellular
141Pr	CD66abce	TET2	Miltenyi	120-014-229	ND	JP042	●	
142Nd	HLA-DR	L243	Ozyme	307651	B219500	JP054	●	
143Nd	CD3	SP34.2	BD	551916	6102787	JP050	●	
144Nd	CD64	10.1	Miltenyi	120-014-229	5170127251	JP056	●	
145Nd	CD8	RPAT8	BD	557084	4357612	JP043	●	
146Nd	IL-6	MQ2.13A5	BD	554543	4283960	JP044		●
147Sm	CD123	7G3	BD	554527	6195661	JP074	●	
148Nd	IL-4	8D48	BD	554515	5134637	JP046		●
149Sm	CD11a	HI111	Miltenyi	120-014-229	5170127255	JP075	●	
150Nd	CD11b	ICRF144	BD	555386	7011599	JP062	●	
151Eu	CD62L	SK11	Miltenyi	120-014-229	5170130321	JP087	●	
152Sm	CD4	L200	BD	550625	6127629	JP057	●	
153Eu	FcεRI	AER37	eBioscience (ThermoFisher Scientific)	14-5899-82	4325555	JP082	●	
154Sm	CD86	IT2.2	BD	555663	5084687	JP045	●	
155Gd	CD125	A14	BD	624084	7095835	JP097	●	
156Gd	CD172a	REA144	Miltenyi	120-014-229	5170127262	JP070	●	
158Gd	IP-10	6D4	Miltenyi	120-014-229	5170127258	JP064		●
159Tb	CD45	D058-1283	BD	552566	6294698	JP098	●	
160Gd	IL-1α	364/3B3	Miltenyi	120-014-229	5170127271	JP066		●
161Dy	CD1c	AF5910	R&D systems (Biotechne)	AF5910	CCXE0216031 and CCXE0216111	JP052	●	
162Dy	IL-12	C8.6	Miltenyi	120-014-229	5170127268	JP071		●
163Dy	CD32	FLI8.26	BD	555447	6077664	JP067	●	
164Dy	IFNα	LT27/295	Miltenyi	120-014-229	5170127269	JP072		●
165Ho	CD39	eBioA1	Biolegend	328002	B215384	JP068	●	
166Er	CCR5	3A9	BD	556041	6224873	JP059	●	
167Er	CD16	3G8	Miltenyi	120-012-311	5170127249	JP083	●	
168Er	CD11c	3.9	Biolegend	301639	B218346	JP060	●	
169Tm	CXCR4	12G5	BD	555971	4055691	JP033	●	
170Er	CD14	M5E2	BD	555396	6112614	JP079	●	
171Yb	IL-8	G265.8	BD	554717	4261555	JP099		●
172Yb	CD23	9P25	Beckman	IMBULK1	16LIQ602	JP065	●	
173Yb	CD141	1A4	Fluidigm	3173002B	2351601	N/A	●	
174Yb	CD20	2H7	BD	556631	6357603	JP061	●	
175Lu	CCR7	G043H7	Miltenyi	120-014-229	5170130220	JP085	●	
176Yb	CADM1	3E1	Clinisciences MBL	CM004-3	039	JP053	●	

Supplementary Table 3 – Antibody panel for mass cytometry. Antibody target, clone, supplier, catalog and lot numbers, and internal batch number after conjugation, and associated metal are indicated as well as whether the antibody was used intra- or extra-cellularly (ND, not defined).

<table>
<tr><th>Kinetic family</th><th>Phenotypic family</th><th>Cell population</th><th>Kinetic Pattern</th></tr>
<tr><td>III</td><td>Granulocytes 4 and 7
Monocytes-DCs 26 and 33</td><td rowspan="6">Eosinophils
Diversely activated cDCs, including cDC1, diversely activated monocytes, and uncharacterized APCs</td><td rowspan="6">Heterogeneous or no response</td></tr>
<tr><td>IX</td><td>Monocytes-DC 20 and 23</td></tr>
<tr><td>X</td><td>Monocytes-DC 24</td></tr>
<tr><td>XI</td><td>Monocytes-DC 25, 31, 35 and 36</td></tr>
<tr><td>XII</td><td>Monocytes-DC 29 and 30</td></tr>
<tr><td>XIII</td><td>Monocytes-DC 34</td></tr>
<tr><td>I</td><td>Granulocytes 1 and 9</td><td rowspan="5">Diversely activated neutrophils including CD14+ neutrophils
Diversely activated monocytes</td><td rowspan="5">Similar response to prime and early boost</td></tr>
<tr><td>II</td><td>Granulocytes 2, 3, 8 and 10
Monocytes-DCs 22 and 37</td></tr>
<tr><td>V</td><td>Granulocytes 6 and 13
Monocytes-DC 17 and 18</td></tr>
<tr><td>VI</td><td>Granulocytes 14
Monocytes-DC 32</td></tr>
<tr><td>VIII</td><td>Granulocytes 16</td></tr>
<tr><td>IV</td><td>Granulocytes 5, 11 and 12</td><td rowspan="2">Eosinophils, basophils and CD86+ activated neutrophils
Diversely activated monocytes, non-classical monocytes / inflammatory cDCs, and pDCs</td><td rowspan="2">Distinct response to prime and early boost</td></tr>
<tr><td>VII</td><td>Granulocytes 15
Monocytes-DCs 19, 21, 27 and 28</td></tr>
</table>

Supplementary Table 4 – Correspondence between kinetic and phenotypic families. For each kinetic family, its composition in terms of phenotypic families are indicated. Moreover, its main cell populations, phenotypes, and kinetic pattern are provided (as classified in **Figure 5**).

Kinetic Family	H6PP vs H0PP	D1PP vs H0PP	D3PP vs H0PP	D8PP vs H0PP	H0PB vs H0PP	H6PB vs H0PP	D1PB vs H0PP	D3PB vs H0PP	D8PB vs H0PP	D14PB vs H0PP	H6PP vs H6PB	D1PP vs D1PB	D3PP vs D3PB	D8PP vs D8PB	D14PP vs D14PB	AUC PP vs AUC PB
I	0,0476	0,0952	0,9048	1,0000	0,2698	0,0238	0,3889	0,5556	0,2460	0,7857	0,9048	0,4762	0,4603	0,2778	0,5079	1,0000
II	0,0159	0,0079	0,2698	1,0000	0,4603	0,0079	0,0159	0,4206	0,4444	0,6270	0,1032	0,0238	0,0476	0,5317	0,6667	0,7222
III	0,0556	0,1349	0,8651	0,8571	0,6825	0,1905	0,7778	0,8571	0,2540	0,6111	0,2540	0,0397	1,0000	0,4048	1,0000	0,3810
IV	0,0079	0,0079	0,7619	0,3095	0,1032	0,0159	0,6032	0,1032	0,0079	0,9048	0,4206	0,0159	0,1190	0,0079	0,2143	0,0079
V	0,0556	0,8333	0,9444	1,0000	0,1746	0,8413	0,0079	0,7937	0,0714	0,7381	0,0556	0,1667	0,8095	0,1032	0,4841	0,1587
VI	0,5635	0,0159	0,0873	1,0000	0,8016	0,0079	0,0079	0,6270	0,5873	0,7222	0,0079	0,0238	0,0873	0,5556	0,7222	0,0794
VII	0,0079	0,0079	0,0238	0,5952	0,4683	0,0079	0,0079	0,6429	1,0000	0,6429	0,6825	0,0079	0,0476	0,6270	0,9365	0,0159
VIII	0,0397	0,4286	0,0873	0,0952	0,1270	0,1270	0,0397	0,1508	0,2937	0,0238	0,3968	0,7698	0,9048	0,7698	0,5952	1,0000
IX	0,0714	0,0556	0,5397	0,8492	1,0000	0,5397	0,2460	0,5397	1,0000	0,8968	0,2460	0,0159	0,7698	0,8810	0,7222	0,2460
X	0,4444	0,2698	0,3175	0,9286	0,8968	0,6825	0,1349	0,0556	0,9365	0,6825	0,1984	0,0635	0,7857	1,0000	0,7619	0,9762
XI	0,7143	0,7460	0,9127	0,9206	1,0000	0,3175	0,1508	0,6349	1,0000	1,0000	0,5000	0,4206	0,8810	0,9206	1,0000	0,7698
XII	0,6270	0,6429	0,8889	0,8571	0,6587	0,8333	0,2540	0,7778	0,8810	0,7222	0,5476	0,0635	0,4603	0,5317	1,0000	0,2619
XIII	0,6111	0,3651	0,4444	0,3810	0,4921	0,5714	0,6111	0,4365	1,0000	0,5635	0,7857	0,0873	0,9365	0,3571	1,0000	0,5000

Supplementary Table 5 – Statistics for kinetic family cell abundance. P-values of the permutation tests are given for the indicated comparisons. Red indicate significant differences, $p < 0.05$.

	BD554D	BT145	CBL004	CC840	CCB116	ALL
**D-27PP**	379 216	542 145	426 000	835 039	453 826	2 636 226
**D-19PP**	572 958	514 273	649 982	516 674	240 091	2 493 978
**H0PP**	485 631	549 104	387 036	830 053	620 959	2 872 783
**H6PP**	470 642	670 857	568 345	772 846	621 498	3 104 188
**D1PP**	632 618	653 277	469 087	838 483	752 279	3 345 744
**D3PP**	796 397	363 416	762 792	677 265	587 500	3 187 370
**D8PP**	822 189	659 918	495 605	759 876	459 273	3 196 861
**H0PB**	860 727	778 709	642 882	657 108	545 693	3 485 119
**H6PB**	697 280	759 853	821 341	676 713	767 993	3 723 180
**D1PB**	667 939	598 244	861 405	670 555	452 805	3 250 948
**D3PB**	447 317	736 728	96 283	674 066	718 784	2 673 178
**D8PB**	242 058	327 046	593 431	291 948	519 604	1 974 087
**D14PB**	536 453	172 437	321 208	499 992	474 893	2 004 983
**D28PB**	564 540	396 758	554 592	1 035 383	623 748	3 175 021
**TOTAL**	8 175 965	7 722 765	7 649 989	9 736 001	7 838 946	41 123 666

Supplementary Table 6 – Numbers of cells per sample. The number of events acquired with the CyTOF is indicated for each sample.

Markers	Number of non-uniform clusters	Percentage of non-uniform clusters
CD32	24	3,00
CD4	18	2,25
CD125	14	1,75
CADM1	14	1,75
CD16	12	1,50
CD123	11	1,38
CD11a	10	1,25
HLA-DR	9	1,13
CD3	9	1,13
FcεRI	8	1,00
CD86	8	1,00
CD11c	8	1,00
CD8	7	0,88
CD14	7	0,88
CD45	6	0,75
CCR5	6	0,75
CD23	6	0,75
CD141	6	0,75
CD11b	5	0,63
CD39	5	0,63
CCR7	5	0,63
CD172a	4	0,50
CD66	3	0,38
CD62L	3	0,38
CD1c	3	0,38
CXCR4	3	0,38
CD64	2	0,25
CD20	2	0,25

Supplementary Table 7 – Uniformity of markers across clusters. For each marker, the numbers, and percentages of non-uniform clusters are indicated.
